# Role of influenza A virus NP acetylation on viral growth and replication

**DOI:** 10.1038/s41467-017-01112-3

**Published:** 2017-11-02

**Authors:** Sebastian Giese, Kevin Ciminski, Hardin Bolte, Étori Aguiar Moreira, Seema Lakdawala, Zehan Hu, Quinnlan David, Larissa Kolesnikova, Veronika Götz, Yongxu Zhao, Jörn Dengjel, Y. Eugene Chin, Ke Xu, Martin Schwemmle

**Affiliations:** 1 Institute of Virology, Medical Center University of Freiburg, 79104 Freiburg, Germany; 2 Faculty of Medicine, University of Freiburg, 79104 Freiburg, Germany; 3 Spemann Graduate School of Biology and Medicine, University of Freiburg, 79104 Freiburg, Germany; 4 Faculty of Biology, University of Freiburg, 79104 Freiburg, Germany; 5 Department of Microbiology and Molecular Genetics, University of Pittsburgh School of Medicine, Pittsburgh, PA 15217 USA; 6 Department of Dermatology, Medical Center, University of Freiburg, Freiburg, 79110 Germany; 7 Department of Biology, University of Fribourg, Fribourg, 79110 Switzerland; 8 Institute of Virology, Philipps-Universität Marburg, 35043 Marburg, Germany; 9 Institute of Health Sciences, Shanghai Institutes for Biological Sciences, Chinese Academy of Sciences, Shanghai, 200031 China; 10 Key Laboratory of Molecular Virology & Immunology, Institut Pasteur of Shanghai, Shanghai Institutes for Biological Sciences, Chinese Academy of Sciences, Shanghai, 200031 China; 11 Present Address: Friedrich Miescher Institute for Biomedical Research, Maulbeerstrasse 66, 4058 Basel, Switzerland

## Abstract

Lysine acetylation is a post-translational modification known to regulate protein functions. Here we identify several acetylation sites of the influenza A virus nucleoprotein (NP), including the lysine residues K77, K113 and K229. Viral growth of mutant virus encoding K229R, mimicking a non-acetylated NP lysine residue, is severely impaired compared to wildtype or the mutant viruses encoding K77R or K113R. This attenuation is not the result of decreased polymerase activity, altered protein expression or disordered vRNP co-segregation but rather caused by impaired particle release. Interestingly, release deficiency is also observed mimicking constant acetylation at this site (K229Q), whereas virus encoding NP-K113Q could not be generated. However, mimicking NP hyper-acetylation at K77 and K229 severely diminishes viral polymerase activity, while mimicking NP hypo-acetylation at these sites has no effect on viral replication. These results suggest that NP acetylation at K77, K113 and K229 impacts multiple steps in viral replication of influenza A viruses.

## Abstract (web summary)

Post-translational modifications of influenza A virus proteins can regulate virus replication, but the effect of nucleoprotein (NP) acetylation is not known. Here, Giese et al. identify four NP lysine residues that are acetylated in infected cells and study their role in polymerase activity and virion release.

## Introduction

The influenza A virus (IAV) genome is composed of eight RNA genome segments (vRNAs) of negative polarity^1^. Each vRNA segment is encapsidated by multiple copies of NP and terminally bound by the viral polymerase subunits PB2, PB1 and PA forming the viral ribonucleoprotein (vRNP) complex^1^. The RNA genome of IAV comprises about 13,600 nucleotides encoding for up to 17 viral proteins^2^. Many of these proteins are multifunctional, playing diverse roles at different stages of the virus infection cycle^3–6^. These include NP, which is not only essential for viral RNA replication and transcription^5^, but also a prerequisite for nuclear transport of vRNPs and packaging of viral genomes into budding viral particles^6–9^. To fulfill these functions in a temporal and spatial manner, NP interacts with a broad spectrum of viral and cellular factors^1, 10, 11^. Furthermore, there is an increasing body of evidence that NP exploits the host’s co- and post-translational modification machinery to regulate its functionality. Recently, it was shown that phosphorylation of NP prevents premature NP oligomerization, thereby allowing its uptake at the elongating chain of newly synthesized genomic RNA^12, 13^. SUMOylation of NP was further shown to be required for the intracellular trafficking of NP^14^. Finally, ubiquitination of NP is believed to regulate viral replication by either increasing (ubiquitination) or decreasing (de-ubiquitination) its binding activity to nascent complementary RNA (cRNA)^15^.

Intriguingly, the architecture of a vRNP complex is similar to that of a nucleosome^16, 17^. Both encapsidated vRNA and cellular DNA are organized into an antiparallel double helix containing a major and minor groove^17, 18^. The positively charged cellular histone molecules are known to bind DNA via the negatively charged phosphate backbone^19^. Similarly, various basic residues in NP convey the interaction with the vRNA phosphate backbone^20, 21^. Epigenetic regulation of histone molecules and thus of the chromatin structure occurs in eukaryotic cells through reversible and coordinated post-translational modifications of histone tails^19, 22^. Commonly, these histone modifications include phosphorylation of serine, threonine and tyrosine residues or acetylation of lysine residues^23, 24^. Currently, no acetylation modifications have been identified for IAV NP. Aside from the recently described phosphorylation sites there are up to 19, partially conserved and surface-exposed lysine (K) residues within the amino acid sequence of NP that might serve as potential targets for acetylation. In general, acetylation is carried out by lysine acetyltransferases (KATs) and reversed by lysine deacetylases (KDACs) to control various cellular and viral protein functions^25, 26^. Hence, we speculated that NP, akin to histones, may be selectively modified by cellular KATs and KDACs to regulate the various functions of NP and vRNPs during the IAV replication cycle.

In this study, we provide evidence that NP is acetylated at specific lysine residues. Mutational analysis of these acetylation sites by mimicking acetylated or non-acetylated lysines suggests that a spatiotemporal balance of the acetylation pattern is required for efficient viral replication.

## Results

### The nucleoprotein harbors several acetylated lysine residues

**Fig. 1:**
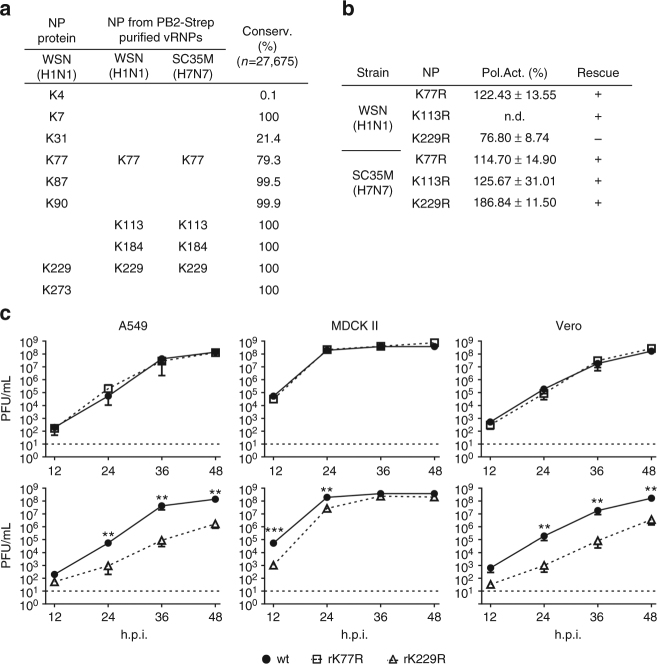
Identification of acetylated NP lysine residues and their role in polymerase activity after substitution with arginine. **a** NP acetylation sites detected by mass spectrometry using transiently expressed NP in HEK293T cells or purified vRNPs from infected A549 cells. Degree of lysine conservation is indicated. **b** Polymerase reconstitution assay in HEK293T cells in the presence of the indicated NP mutants. Polymerase activity is shown as mean and s.d. of at least three independent experiments (n.d., not determined). Plus indicates successful rescue of recombinant viruses encoding acetylation-deficient NP. **c** MDCK II, A549 and Vero cells were infected with the indicated viruses at an MOI of 0.001 and viral growth was monitored 12, 24, 36 and 48 h.p.i. by plaque assay. Dashed lines represent the detection limit. **p < 0.01; ***p < 0.001. Error bars indicate the mean and s.e.m. of at least three independent experiments. Student’s *t*-test was used for two-group comparisons

IAV NP contains up to 19 lysine (K) residues with varying degrees of conservation (Supplementary Fig. 1a). To identify putative acetylation sites, we performed mass spectrometry analysis on purified HA-tagged NP protein of A/WSN/1933 (H1N1), transiently expressed in HEK293T cells (Supplementary Fig. 1b). To achieve efficient NP acetylation, the acetyltransferase CREB-binding protein (CBP) was additionally co-expressed (Supplementary Fig. 1b). Eight different acetylated K residues were identified, including five highly conserved residues at positions 7, 87, 90, 229 and 273 (Fig. 1a and Supplementary Fig. 2a, b). To identify acetylated lysine residues on NP within vRNP complexes, we infected human A549 cells with recombinant WSN encoding Strep-tagged PB2 at a multiplicity of infection (MOI) of 20 for 20 h and subsequently purified vRNPs (Supplementary Fig. 1c) as described previously^27^. Four acetylation sites (K77, K113, K184 and K229) were confirmed by mass spectrometry analysis as present within the purified vRNP complexes (Fig. 1a and Supplementary Fig. 2c). Likewise, acetylated K77, K113, K184 and K229 were also detected in the vRNPs of A/SC35M/1980 (H7N7) in at least four independent vRNP preparations (Fig. 1a and Supplementary Fig. 2c). Since it was shown previously that ubiquitination of K184 regulates polymerase activity^15^ we did not include this amino acid residue in our mutational study. Therefore, to investigate solely NP acetylation upon infection, we chose to further characterize K77, K113 and K229.

### Mimicking non-acetylated lysine residues at K229

To elucidate the influence of NP acetylation on viral polymerase activity, we generated NP mutant proteins of SC35M and WSN with individual K to arginine (R) mutations (NP-K77R, NP-K113R, NP-K229R) with the exception of WSN NP-K113R. This strategy is commonly used to mimic non-acetylated lysine, since an arginine substitution prevents acetylation but preserves charge^28–30^. All NP mutant proteins were expressed at comparable levels (Supplementary Fig. 3a) and supported polymerase activity as assessed by polymerase reconstitution assay in HEK293T cells (Fig. 1b). With the exception of the WSN NP mutant NP-K229R all other K to R mutant proteins resulted in similar or even enhanced polymerase activities compared to wildtype (wt) WSN or SC35M NP, respectively. As expected, we could generate recombinant SC35M mutant viruses encoding NP-K77R, NP-K113R or NP-K229R (Fig. 1b), further designated rK77R, rK113 and rK229R, respectively. However, even though both WSN NP non-acetylation mutants NP-K77R and NP-K229R supported polymerase activity, we already failed to generate recombinant WSN viruses expressing NP-K229R (Fig. 1b). To study the effects at all three NP acetylation sites in the context of viral infections, we narrowed our focus to SC35M mutant viruses for further analysis.

**Fig. 2:**
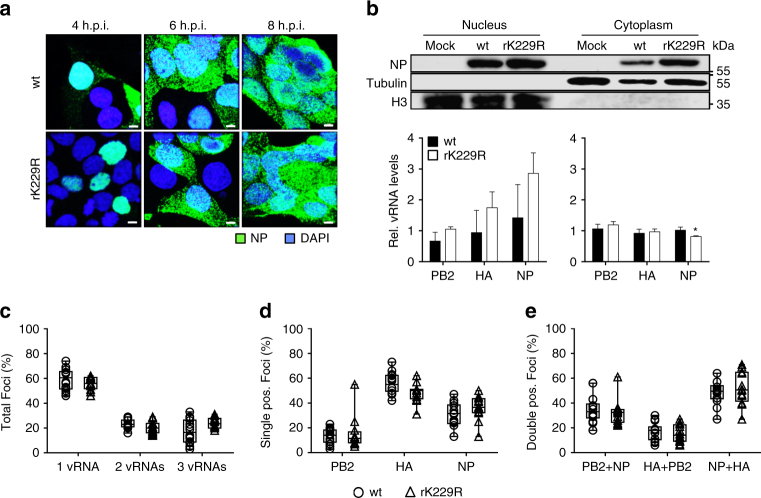
Growth deficiency of NP mutant virus rK229R. **a** Subcellular localization of NP in MDCK II cells infected with the indicated viruses at an MOI of 5 after 4, 6 and 8 h.p.i. Scale bar = 4 µm. **b** Cytoplasmic and nuclear accumulation of viral protein and RNA. MDCK II cells were infected at an MOI of 5 and 6 h.p.i., nuclear and cytoplasmic fractions were obtained. Upper panels show protein levels determined by Western blot analysis. β-Tubulin and histone H3 were used as markers for specific fractionation. Lower panels: relative vRNA transcript levels (PB2, HA, NP) in the nuclear and cytoplasmic fractions determined by quantitative RT–PCR. *p < 0.05. Error bars indicate the mean and s.d. of at least three independent experiments. Student’s *t*-test was used for two-group comparisons. **c**–**e** Fluorescence in situ hybridization to examine co-segregation of vRNAs in wt SC35M and rK229R-infected cells. MDCK II cells were infected at an MOI of 5 for 6 h and vRNAs were subsequently stained with segment-specific and fluorescently labeled probes. **c** Total cytoplasmic foci, positive for either one (HA, NP or PB2), two (HA and NP; HA and PB2; NP and PB2) or three vRNAs (HA, NP and PB2). **d** Composition of cytoplasmic foci positive for only one vRNA. **e** Cytoplasmic foci, positive for two co-segregating vRNAs. Each dot represents the total cytoplasm of a single cell. Depicted are 12–14 infected cells per virus from >4 independent infections. A two-tailed Mann–Whitney test was used for two-group comparisons

In order to characterize the growth properties of NP acetylation-deficient SC35M viruses, A549, MDCK II and Vero cells were infected with rK77R, rK113R or rK229R at an MOI of 0.001. Both rK77R (Fig. 1c) and rK113R (Supplementary Fig. 4), irrespective of the cell line, displayed no growth deficiency compared to wt SC35M, whereas rK229R was significantly attenuated (Fig. 1c). Since mimicking non-acetylation at K229 did not inhibit viral polymerase activity (Fig. 1b), we speculated that NP acetylation at this particular lysine residue might either regulate NP trafficking similar to NP SUMOylation at amino acids K4 and K7^14^, or directly control nuclear export of newly synthesized vRNP complexes. However, we did not observe differences in NP re-localization from the nucleus to the cytoplasm in MDCK II cells infected with rK229R (Fig. 2a) at an MOI of 5 at 4, 6 and 8 h post infection (h.p.i.). A comparable time-dependent re-localization of NP was also observed in A549 cells infected with mutant and wt viruses (Supplementary Fig. 5). Consistently, cellular fractionation to determine NP levels in the nuclear and cytoplasmic fraction, followed by quantitative RT–PCR of three different vRNA segments (PB2, HA and NP) revealed only subtle differences between wt and NP acetylation-deficient mutant rK229R (Fig. 2b).

Next, we examined whether NP acetylation at K229 might be required to regulate vRNP co-segregation^31, 32^ during cytoplasmic trafficking en route to the apical plasma membrane (APM). For this purpose we performed three-color fluorescence in situ hybridization (FISH)^32^, simultaneously visualizing the aforementioned PB2, HA and NP vRNAs in MDCK II cells infected at an MOI of 5 for 6 h. To exclude nuclear vRNAs, we performed DAPI staining, while simultaneous NP protein staining was used to define the cellular plasma membrane. We confirmed robust spectral separation of our three FISH probes, similar to a previous report^32^, using single color controls (Supplementary Fig. 6). Entire cell volumes were imaged using fine *z*-stacks to analyze the three-dimensional cellular distribution of vRNPs. We observed that the composition of vRNA containing cytoplasmic foci was similar between wt and rK229R-infected cells (Fig. 2c). Consistent with a previous report^32^, most foci were positive for one vRNA (wt SC35M: 59.3% and rK229R: 55.9%), whereas foci comprising two (wt SC35M: 23.4% and, rK229R: 20.4%), or all three vRNAs (wt SC35M: 17.4% and rK229R: 23.8%) were found less frequently (Fig. 2c). Single positive foci usually contained HA (wt SC35M: 55.7% and rK229R: 48.3%) or NP (wt SC35M: 30.6% and rK229R: 36.1%) vRNA, while PB2 vRNA containing foci were rarer (wt SC35M: 13.5% and rK229R: 15.5%; Fig. 2d). Foci which were found positive for two vRNAs were used to analyze the impact of NP acetylation on vRNA co-segregation (Fig. 2e). As depicted, mean values are similar for co-segregation of PB2 and NP vRNA (wt SC35M 34.1%; rK229R 32.4%), HA and PB2 vRNA (wt SC35M 17.4%; rK229R 15.4%) or NP and HA vRNA (wt SC35M 48.6%; rK229R 51.9%). Overall, these results suggest that NP acetylation at lysine residue K229 is not required to regulate viral replication, the nuclear export of newly synthesized vRNPs or co-segregation of exported vRNPs in the cytoplasm.

### The NP mutation K229R impairs viral particle release

**Fig. 3:**
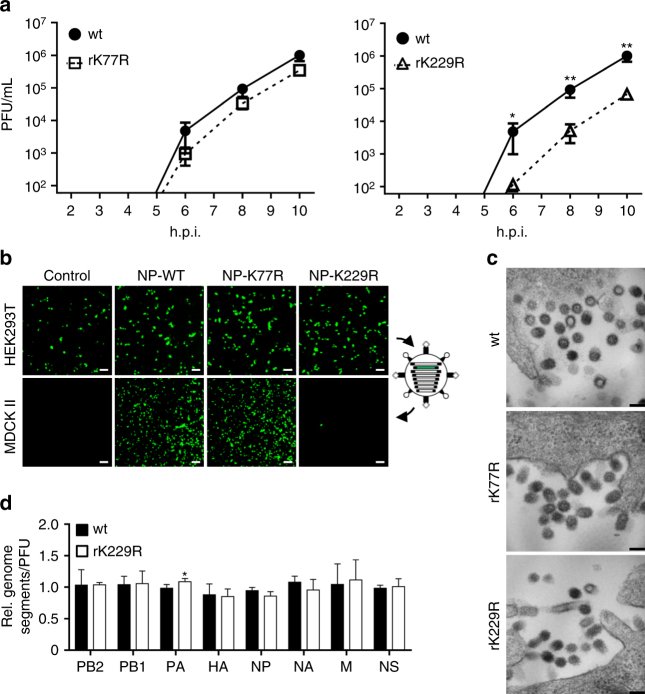
Mimicking non-acetylated K229 of SC35M NP negatively affects particle release but not genome packaging. **a** To determine viral particle release under single cycle growth conditions, infection of MDCK II cells (MOI 10) was synchronized on ice and viral titers were subsequently determined via plaque assay at the indicated time points. Error bars indicate the mean and standard error of the mean (s.e.m) of at least four independent experiments. Student’s *t*-test was used for two-group comparisons. *p < 0.05; **p < 0.01. **b** To investigate genome incorporation mediated by wt or mutant SC35M NP proteins, VLPs were generated in HEK293T cells in the presence of a GFP-encoding reporter minigenome flanked by PB1 packaging sequences together with the remaining 7 genome segments. Cell supernatant containing released VLPs was used to infect MDCK II cells, which were subsequently superinfected with wt SC35M. Scale bar = 100 µm. **c** To analyze viral particle morphology, MDCK II cells were infected (MOI 10), fixed 10 h.p.i. and subjected to electron microscopy. Representative pictures of individual particles are shown. Scale bar = 100 nm. **d** Packaging of genome segments incorporated into viral particles released from MDCK II cells infected with the indicated viruses. Equal amounts of infectious viral particles determined by PFU were analyzed by quantitative RT–PCR. Levels of viral genome transcripts of wt SC35M were set to 1. Error bars indicate the mean and s.d. of at least three independent experiments. *p < 0.05. Student’s *t*-test was used for two-group comparisons

Considering that the NP acetylation-deficient mutant rK229R was severely attenuated in various cell types (Fig. 1c), but revealed no altered protein localization or disordered vRNP co-segregation upon infection (Fig. 2), we hypothesized that NP acetylation at K229 might be required for particle release. To show this, we infected MDCK II cells at an MOI of 10 with the mutant and wt viruses and determined infectious viral particle release at early time points post infection. Mutant rK77R was included as positive control. Under these single cycle growth conditions release of infectious virus was severely reduced in cells infected with rK229R compared to wt or rK77R (Fig. 3a). A comparable block in particle release was also observed after reconstitution of SC35M virus-like particles (VLPs) in HEK293T cells in the presence of the NP acetylation mutant. VLPs were generated containing seven distinct SC35M genome segments (segment 1 and 3–8) together with a vRNA-like eGFP reporter segment flanked by the 3′ and 5′ non-coding and terminal coding regions of the PB1 segment (Supplementary Fig. 3d). As expected from the pronounced activity observed in the polymerase reconstitution assay (Fig. 1b) we detected robust eGFP expression in HEK293T cells for wt SC35M NP, NP-K77R and NP-K229R (Fig. 3b, upper panel). Infection of MDCK II cells with VLPs reconstituted with wt SC35M NP or NP-K77R and subsequent superinfection with wt SC35M resulted in numerous eGFP-expressing cells, indicating successful incorporation of the reporter segment (Fig. 3b, lower panel). However, reconstitution with NP-K229R resulted in fewer VLPs (Supplementary Fig. 3e) and GFP-positive cells (Fig. 3b, lower panel).

To further investigate whether NP acetylation-deficient mutant rK229R would display aberrant particle morphology, we infected MDCK II cells at an MOI of 10 and analyzed released virions by electron microscopy (EM). However, we did not observe grossly altered shape of viral particles between wt SC35M and mutant-infected cells (Fig. 3c). As shown recently^9^, NP amino acid substitutions can cause a genome packaging defect and the release of viral particles which cannot initiate a full replication cycle (semi-infectious particles). To rule out the possibility of a packaging defect, we determined the relative ratio of all eight viral genome segments using quantitative RT–PCR of an equal number of wt SC35M and rK229R infectious viral particles. As depicted, only subtle changes in particle genome content were detected between wt SC35M and the mutant virus rK229R (Fig. 3d). Taken together, these results indicate that the NP mutant protein NP-K229R causes a block in the efficient release of infectious virions.

### Mimicking acetylation at either K113 or K229

**Fig. 4:**
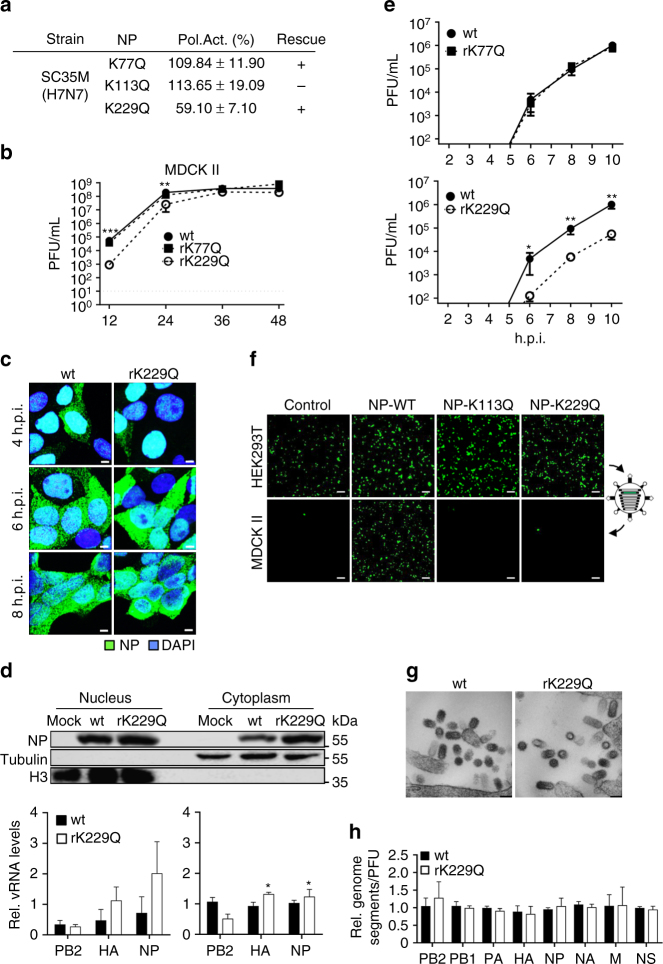
Impact on viral replication and growth mimicking constant NP acetylation. **a** Polymerase reconstitution assay in HEK293T cells in the presence of the indicated NP mutants. Polymerase activity is shown as mean and s.d. of at least three independent experiments. Plus indicates successful rescue of recombinant viruses. **b** MDCK II cells were infected at an MOI of 0.001 and viral growth was monitored at the indicated time points by plaque assay. Dashed lines represent the detection limit. **p < 0.01; ***p < 0.001. Error bars indicate the mean and s.e.m. of at least three independent experiments. **c** Subcellular localization of NP in MDCK II cells infected with the indicated viruses at an MOI of 5 after 4, 6 and 8 h.p.i. Scale bars = 4 µm. **d** NP protein (upper panels) and vRNA transcript (PB2, HA, NP) levels (lower panels) were determined in nuclear and cytoplasmic fractions 6 h.p.i. of MDCK II cells infected at an MOI of 5 by Western blot analysis and quantitative RT–PCR, respectively. β-Tubulin and histone H3 were used as marker for specific fractionation. Error bars indicate the mean and s.d. of at least three independent experiments. *p < 0.05. **e** Infection of MDCK II cells (MOI 10) was synchronized on ice and viral titers were subsequently determined via plaque assay. Error bars indicate the mean and standard error of the mean (s.e.m) of at least four independent experiments. Student’s *t*-test was used for two-group comparisons. *p < 0.05; **p < 0.01. **f** VLPs were generated in HEK293T cells in the presence of a GFP-encoding reporter segment with the remaining seven genome segments. VLPs released into the cell supernatant were used to infect MDCK II cells, which were subsequently superinfected with SC35M. Scale bar = 100 µm. **g** MDCK II cells were infected (MOI 10), fixed 10 h.p.i. and subjected to electron microscopy. Representative pictures are shown. Scale bar = 100 nm. **h** Relative amount of genome segments incorporated into viral particles. Equal amounts of infectious viral particles determined by PFU were analyzed by quantitative RT–PCR. Level of wt SC35M genome transcripts were set to 1. Error bars indicate the mean and s.d. of at least three independent experiments. Student’s *t*-test was used for two-group comparisons

Next, we generated NP variants mimicking constant acetylation by substituting the respective lysine residue with neutrally charged glutamine (Q), a known acetylation-mimic^28, 29^. As shown in Fig. 4a, both SC35M-NP-K77Q and SC35M-NP-K113Q supported polymerase activity to comparable levels as wt NP, whereas the polymerase activity in the presence of SC35M-NP-K229Q was reduced to 59%, despite comparable protein expression (Supplementary Fig. 3b). Intriguingly, though we observed wt-like activity of NP-K113Q in the polymerase reconstitution assay, we could not generate SC35M virus encoding this mutant protein (Fig. 4a). However, we were able to rescue both NP-K77Q (rK77Q) and NP-K229Q (rK229Q) in the context of SC35M. Compared to wt SC35M and rK77Q, viral growth of rK229Q was significantly impaired in MDCK II cells infected at an MOI of 0.001 at 12 and 24 h.p.i. (Fig. 4b) and severe attenuation of rK229Q was also observed in A549 and Vero cells (Supplementary Fig. 7a). MDCK II cells (Fig. 4c) or A549 cells (Supplementary Fig. 7b) infected with rK229Q revealed wt-like subcellular re-localization of NP. Consistently, fractionation of MDCK II cells infected with either wt SC35M or rK229Q at an MOI of 5 revealed comparable NP protein and vRNA levels (PB2, HA and NP) in the nuclear and cytoplasmic fraction (Fig. 4d) at 6 h.p.i. By restricting viral replication to a single round of infection, less infectious viral particles were detected for rK229Q compared to rK77Q and wt infected cells (Fig. 4e), suggesting that the NP mutation K229Q might also diminish release of viral particles as the NP mutation K229R (Fig. 3). As expected, reconstitution of VLPs comprising NP-K229Q in the presence of eight genome segments was reduced compared to wt SC35M NP (Fig. 4f and Supplementary Fig. 3e). Consistent with the failure to rescue infectious virus (Fig. 4a), we could not reconstitute VLPs in the presence of NP-K113Q (Fig. 4f), however, succeeded as expected with NP-K113R (Supplementary Fig. 7c). As shown for the NP acetylation-deficient mutant rK229R, morphology of released viral particles (Fig. 4g) and their genome content (Fig. 4h) was not affected by K229Q substitution.

In summary, based on our mimics acetylation or no acetylation at either K113 or K229 resulted in different outcomes. Mimicking either acetylation or no acetylation at K229 impaired particle release. In contrast, mimicking acetylation at K113 completely abrogated viral growth at a stage beyond viral replication, whereas mimicking non-acetylated K113 had no detectable effect on both polymerase activity and viral growth.

### NP hyper-acetylation at K77 and K229 disturbs vRNP formation

**Fig. 5:**
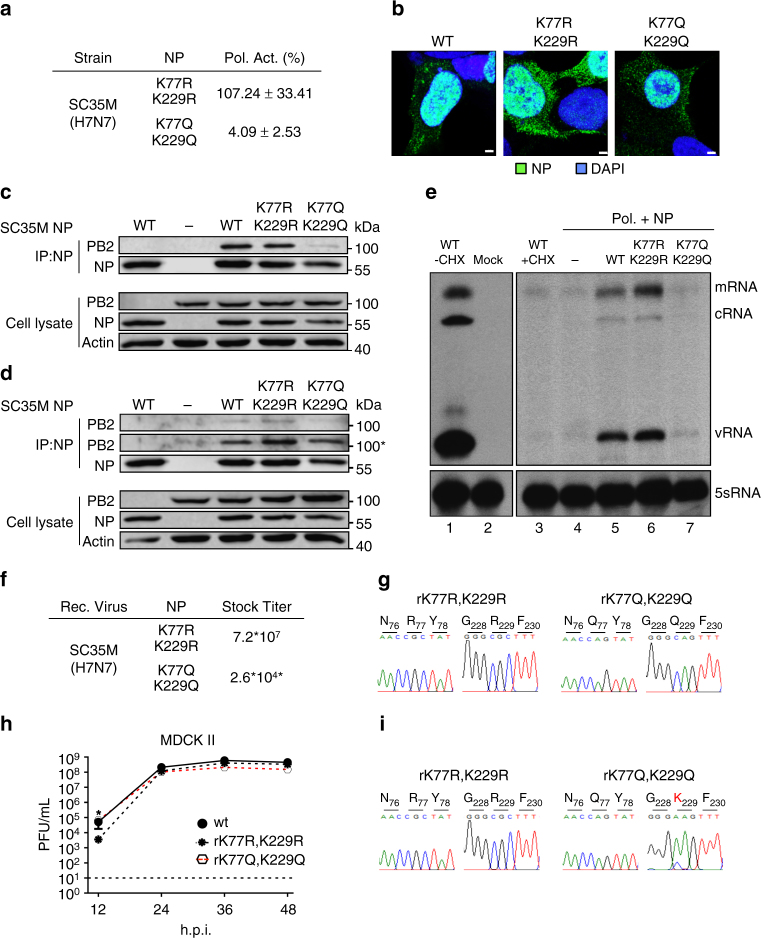
Mimicking concurrent acetylation at K77 and K229 of SC35M NP is not tolerated by the virus. **a** Polymerase reconstitution assay in HEK293T cells in the presence of the indicated NP mutants. Polymerase activity is shown as mean and s.d. of at least three independent experiments. **b** Nuclear localization of the respective NP mutants after transient expression in HEK293T cells. Scale bar = 2 µm. **c**, **d** Binding of PB2 to SC35M NP mutants in presence **c** or absence **d** of vRNA. After reconstitution of the polymerase complex in HEK293T cells for 24 h, cells were lysed and NP was immunoprecipitated (IP) using an NP-specific monoclonal antibody. Protein levels after IP and in the cell extract were determined by Western blot. Actin serves as loading control. (Asterisks) panel with increased exposure to visualize residual PB2 binding. **e** Following transfection of HEK293T cells with control plasmid (empty vector) the polymerase complex and NP (either wt or indicated mutants), cells were infected at an MOI of 5 in presence of cycloheximide (CXH). 6 h.p.i. RNA was isolated and viral PB2 transcripts were determined by primer extension analysis. Cellular 5sRNA serves as loading control. Right upper panel represents an enhanced exposure to visualize the viral transcript levels in the presence of CXH. **f** Stock titers of SC35M virus mutants observed after rescue and one round of plaque purification. Viral titers (PFU/mL) were determined by plaque assay at 48 h.p.i. (Asterisks) Titers for rK77Q,K229Q were determined at 72 h.p.i. **g** To confirm the respective mutations in both stock viruses, viral RNA was isolated and reverse transcribed using segment-specific primers. **h** MDCK II cells were infected with the indicated viruses at an MOI of 0.001 and viral growth was monitored 12, 24, 36 and 48 h.p.i. by plaque assay. Dashed lines represent the detection limit. *p < 0.05. Error bars indicate the mean and s.e.m. of at least three independent experiments. Student’s *t*-test was used for two-group comparisons. **i** To determine the NP sequences, viral RNA was isolated from viral particles 48 h.p.i. and reverse transcribed using segment-specific primers

Considering, that various NP lysine residues might be concurrently acetylated, we generated SC35M NP mutant proteins harboring either two K to R or K to Q substitutions at position 77 and 229 and tested these mutants upon polymerase reconstitution in HEK293T cells. We excluded K113 from this analysis, since the NP mutation K113Q abrogates viral growth, impeding potential further analysis in the context of infectious virus. Polymerase reconstitution with the NP mutant NP-K77R,K229R mimicking constant non-acetylation resulted in 107% polymerase activity, compared to wt SC35M NP (Fig. 5a). In contrast, mimicking constant acetylation (NP-K77Q,K229Q) severely diminished polymerase activity to below 5% (Fig. 5a), despite comparable NP expression levels to wt SC35M NP (Supplementary Fig. 3c). However, similar nuclear accumulation of wt SC35M NP, NP-K77R,K229R and NP-K77Q,K229Q was observed after transient expression of these proteins in HEK293T cells (Fig. 5b). Consistent with the aforementioned activity in the polymerase reconstitution assay (Fig. 5a), immunoprecipitation of wt SC35M NP or NP-K77R,K229R using NP-specific antibodies resulted in the co-immunoprecipitation of PB2, whereas only residual PB2 levels were observed after immunoprecipitation of NP-K77Q,K229Q (Fig. 5c). Interestingly, in a parallel polymerase reconstitution experiment in which the viral minigenome was excluded (no functional vRNP formation) lower levels of PB2 were co-immunoprecipitated with wt SC35M NP, NP-K77R,K229R and NP-K77Q,K229Q (Fig. 5d, upper panel). However, enhanced exposure time revealed that overall comparable PB2 levels were co-immunoprecipitated (Fig. 5d, second upper panel). Hence, decreased polymerase activity in presence of NP-K77Q,K229Q is not due to impaired PB2 binding but a step in the viral replication. Considering that K77 and K229 are localized in the proximity of the NP RNA binding groove^20, 21^ we speculated that acetylation might alter its ability to encapsidate genomic RNA intermediates such as cRNA. To show this, we infected HEK293T cells with SC35M wt virus at an MOI of 5 for 6 h in the presence of cycloheximide to prevent mRNA translation, only allowing primary transcription of mRNA and cRNA from the incoming vRNA. Since cRNA is rapidly degraded, visualization of this primary transcript requires its stabilization by overexpression of the polymerase subunits and NP prior infection and cycloheximide treatment^3, 5^. As shown in Fig. 5e, cycloheximide treatment resulted in detectable but low levels of incoming vRNA and the accumulation of primary mRNA transcripts (Fig. 5e, lane 3), whereas in the absence of cycloheximide all viral RNA species, including cRNA, were detected (Fig. 5e, lane 1). As described previously^3, 5^, prior overexpression of the polymerase complex and NP wt resulted in traceable levels of cRNA and consequentially enhanced v- and mRNA levels (Fig. 5e, lane 5). Enhanced levels of viral transcripts were also obtained with NP-K77R,K229R (Fig. 5e, lane 6). In contrast, in the presence of NP-K77Q,K229Q fewer cRNA and vRNA levels were observed (Fig. 5e, lane 7). Together these results suggest that NP-K77Q,K229Q fails to efficiently stabilize cRNA, thereby abrogating the viral replication process.

Next we tried to rescue SC35M mutant viruses encoding either NP-K77R,K229R (rK77R,K229R) or NP-K77Q,K229Q (rK77Q,K229Q). Astonishingly, both mutant viruses could be generated by reverse genetics, although rK77Q,K229Q revealed a virus stock titer that was 2770 fold lower than r77R,K229R (Fig. 5f). Sanger sequencing of both stock viruses confirmed that the respective mutations were indeed present within NP (Fig. 5g). To characterize viral growth, we infected MDCK II cells at an MOI of 0.001, determining viral titers at 12, 24, 36 and 48 h.p.i. In line with the results obtained for mutant virus rK229R, the double mutant rK77R,K229R showed impaired viral growth in MDCK II cells at early time points of infection (Fig. 5h). Strikingly, no attenuation was observed with rK77Q,K229Q (Fig. 5h). Sequencing of both mutants at 48 h.p.i. revealed that the two arginine substitutions of rK77R,K229R were maintained, whereas in cells infected with rK77Q,K229Q, an escape mutation emerged, restoring a wt-like lysine residue at position 229 (Fig. 5i).

Taken together, in contrast to mimicking NP hypo-acetylation, mimicking constant acetylation at amino acid residues 77 and 229 of NP resulted in impaired viral replication possibly due to a failure to efficiently stabilize replication intermediates such as cRNA.

## Discussion

In virus-infected cells several lysine residues of NP, including K77, K113 and K229 were identified to be acetylated. Using a mutational approach in which we mimicked non-acetylated lysine residues by substitution with arginine (R), the mutation K229R in NP of SC35M was found to impair viral growth in multiple cell lines, whereas the replacement of the lysine residues at positions 77 and 113 with arginine had no detectable effect. In comparison to wt SC35M, the mutant virus encoding NP-K229R showed similar viral polymerase activity, time-dependent accumulation of vRNPs in the cytoplasm and co-segregation of distinct cytoplasmic vRNPs. However, single cycle growth kinetics suggested that release of infectious viral particles was severely diminished, although this deficiency did not induce erroneous particle genome content. Mimicking a single constitutively acetylated lysine residue by substituting K229 with glutamine (K229Q) revealed a comparable defect in particle release, whereas the NP mutation K77Q displayed no effect. Intriguingly, the NP mutation K113Q did not allow the generation of viable virus, despite unimpaired polymerase activity. Furthermore, mimicking constitutive acetylation of both NP lysine residues K77 and K229 severely diminished viral replication by the inability to initiate amplification of vRNA copies, whereas mimicking non-acetylated lysines at these positions had no effect on viral polymerase activity.

Mutational approaches that aim to mimic acetylated and non-acetylated lysine residues by arginine or glutamine substitutions have been successfully used in numerous studies to gain insight into the functional role of protein acetylation^28–30, 33^. This approach is based on the assumption that the major purpose of acetylation is to neutralize the positive charge of lysine moieties. As a consequence, a conversion to glutamine should induce a different phenotype than an arginine substitution. However, since the SC35M viruses encoding either K to R (rK229R) or K to Q (rK229Q) mutations displayed similar phenotypes, it cannot be ruled out that the observed impaired particle release is independent of acetylation and rather caused by structural alterations due to mutation of K229. Such a phenomenon has been previously suggested for acetylatable lysine residues^34, 35^. Alternatively, transient acetylation spanning the vRNP could be required for efficient particle release since each vRNP is composed of around 35–95 nucleoproteins, of which only a fraction are presumably acetylated at position K229. Thus, shifting to either acetylation (Q substitution) or non-acetylation (R substitution) might cause significant changes in the overall charge of the vRNPs and thereby compromise important functions in the budding process of viral particles. At which step viral particle release is blocked in cells infected with the mutant viruses mimicking acetylated or non-acetylated lysine residues at position 229 remains to be shown. As described by others, mutations at WSN NP residues 214, 217 and 253 are capable of changing virion morphology from spherical to filamentous particles, due to altered NP-M1 binding^36^. However, particles released from cells infected with the acetylation mutants show comparable virion morphology as wt virus particles, suggesting that impaired NP-M1 binding may not be the cause for the particle release deficit.

Competition between acetylation and ubiquitination at lysine residues is known to occur^33^, complicating mutational approaches. Indeed, recent studies by Lin et al.^37^ revealed that 10 out of 19 lysine residues are ubiquitinated after transient overexpression of WSN NP, including K77, K113 and K229. However, although it remains a possibility, NP has not yet been shown to be ubiquitinated at these sites in infected cells. Still, it cannot be formally excluded that a fraction of K77, K113 and K229 is present with other post-translational modifications than acetylation. Nevertheless, mutations of K77 to either R or Q or K113 to R had no apparent effect on viral replication, implying that ubiquitination at these particular sites can most likely be neglected. Provided that SC35M K229 is indeed ubiquitinated in virus-infected cells, loss of ubiquitination at this site due to mutations may contribute to the observed defect in viral growth and particle release.

In contrast to the effect observed upon the amino acid substitutions at K229, mutation of K113 in NP to arginine resulted in a dramatically different phenotype as that of glutamine. Despite the mutation K113R having no detectable effect on polymerase activity or viral growth, rescue of NP-K113Q virus was not possible. This strongly suggests that acetylation of K113 does in fact affect a later stage in the viral life cycle past viral replication. Notably, a reciprocal interplay between lysine acetyltransferases (KATs) and lysine deacetylases (KDACs) is required to regulate lysine acetylation of histones^38^. Identifying which of the 17 KATs or 18 KDACs^25^ coordinate acetylation/deacetylation at the aforementioned lysine residues is necessary to further elucidate the regulatory mechanisms of NP acetylation. However, this could prove to be a comprehensive task due to the functional redundancies amongst KATs or KDACs and their vast substrate diversity.

Contrary to the single amino acid mutational approach, mimicking acetylation and non-acetylation at both NP lysine residues K77 and K229 revealed strongly opposing effects on viral replication. Whereas the NP mutations K77Q and K229Q severely diminished viral replication, K77R and K229R had no effect in this regard, suggesting that simultaneous acetylation at these sites specifically interferes with a certain step in the viral life cycle. The block in viral growth mediated by NP-K77Q,K229Q appears to be at the stage of viral replication. As shown by the cRNA stabilization assay, only reduced levels of cRNA are observed in cells infected with SC35M (in the presence of cycloheximide) and prior expression of the polymerase complex and NP-K77Q,K229Q. This indicates that NP-K77Q,K229Q at least fails to properly encapsidate cRNA transcripts whereas PB2 binding seems not to be affected. Since both K77 and K229 are found within close proximity of the known vRNA-binding groove^20, 21^, we speculate that mimicking hyper-acetylation at K77 and K229 might adversely affect its overall binding capacity to the negatively charged cRNA phosphate backbone, thereby preventing proper encapsidation. However, it should be noted that the mass spectrometry data doesn’t allow to conclude that both lysine residues are simultaneously acetylated on the same NP molecule in infected cells. Thus, biological importance of contemporaneous acetylation at K77 and K229 remains to be shown.

In summary, using a mutational approach we provide evidence that acetylation of highly conserved lysine residues might regulate specific functions of NP in the viral life cycle of influenza A viruses, including viral replication.

## Methods

### Cell lines

Vero (ATCC; #CCL-81), A549 (ATTC; # CCL-185), MDCK II (Merck; #00062107) and HEK293T cells (ATTC; CRL-3216) were cultured in Dulbecco’s modified Eagle’s medium (DMEM, Gibco, Thermo Scientific) containing 10% fetal calf serum (FCS), 100 U Penicillin and 100 mg Streptomycin per ml at 37 °C and 5% CO_2_.

### Plasmids

The pHW2000-based rescue system to generate recombinant WSN and SC35M viruses and pCAGGs plasmids encoding A/WSN/33 and A/SC35M/1980 proteins have been previously described^3, 9, 14, 39^. Plasmids encoding NP with mutations (K to R or K to Q) at amino acid position K77, K113 and K229 were generated using site-specific mutagenesis primers. The pHW400_PB1_eGFP reporter minigenome has been generated as previously described^9^. The GFP protein coding sequence was flanked by assembly PCR with the 5′ (nt 1–90) and 3′ (nt 2259–2341) termini of PB1 and cloned into the pHW400 vector allowing polI-driven expression of the reporter minigenome. The pHW400 was generated by removing the polII promoter and terminator from the pHW2000 rescue vector as described^9^. The human C-terminal HA-tagged CBP (CBP-HA) gene (NM_004380.2) was cloned into pcDNA3.0 (ThermoFischer).

### Virus strains and infections

Recombinant viruses WSN-PB2-Strep, WSN, SC35M and SC35M-PB2-Strep were generated as previously described^27^. All recombinant viruses were plaque purified and viral titers were determined by plaque assay on MDCK II cells. NP mutations at the respective positions were confirmed by sequencing. If not indicated otherwise, cells were washed with PBS (0.2% BSA) and infection was performed in infection media (DMEM containing 0.2% BSA, 100 U Penicillin and 100 mg Streptomycin per ml). For single cycle infections, cells were incubated with viruses in infection media on ice. After 45 min, cells were incubated for 7 min at 37 °C, washed with PBS, incubated in PBS (pH = 2) for 30 s, washed with infection media and further incubated at 37 °C in infection media for the indicated time points.

### Subcellular fractionation

MDCK II cells were washed in PBS and infected with the respective virus (MOI 5) at room temperature in infection media. After 1 h, inoculum was removed and cells were washed 3 times with PBS (0.2% BSA) and further incubated in infection media at 37 °C and 5% CO_2_ for 6 h. Afterwards, cells were washed and pelleted (600 *g*, 5 min, 4 °C) in ice-cold PBS. Nuclear and cytoplasmic fractionation was performed with the Nuclear/Cytosol Fractionation Kit (BioVision) according to manufacturer’s protocol. Samples were either subjected to Western blot analysis or total RNA extraction using Direct-zol RNA MiniPrep Kit (Zymo Research).

### Western Blot analysis

Proteins from cell lysates were separated by sodium dodecyl sulfate polyacrylamide gel electrophoresis (SDS–PAGE) and subjected to Western blot analysis. NP, actin, tubulin or histone H3 levels were determined using specific antibodies against NP (Gene Tex, GTX125989, 1:1000), actin (Sigma-Adlrich, A3853; 1 : 1000), tubulin (Sigma-Aldrich, T4062; 1 : 1000) and histone H3 (Abcam, AB1791; 1 : 1000), respectively. Monoclonal PB2 (1 : 200) antibody was kindly provided by Dr. Georg Kochs. NP acetylation was determined using a pan-specific lysine acetyl antibody (Immunechem, ICP0380; 1 : 1000). Anti-HA antibody (Sigma-Aldrich, H6908; 1 : 2000) was used to detect HA-tagged proteins. Primary antibodies were detected using Peroxidase-conjugated secondary antibodies (Jackson ImmunoResearch, 1 : 5000). Uncropped scans of the Western blots can be found in Supplementary Fig. 8.

### Immunofluorescence

MDCK II, A549 or HEK293T cells seeded onto glass plates were either infected with the respective virus (MOI 5) at room temperature for 1 h or transfected (each 250 ng). Viral supernatant was removed and cells were washed three times in PBS (0.2% BSA) and further incubated in infection media at 37 °C and 5% CO_2_. At the indicated time points, cells were fixed using 4% paraformaldehyde (PFA) in PBS for 15 min, washed three times with PBS and permeabilized with 0.5% Triton X-100 in PBS for 5 min. Viral proteins were stained with antibodies against NP (ATCC: H16-L10-4R5) at a dilution of 1:1500, for 1 h at room temperature. Upon washing, cells were stained using a corresponding secondary anti-mouse IgG coupled to AlexaFluor 488 (Jackson ImmunoResearch, 1:500) for 1 h. Finally, cells were washed and nuclei were stained for 5 min using 4′,6-diamidino-2-phenylindole (DAPI) at a dilution of 1 : 10,000 in PBS. Glass slides were mounted and examined on a LEICA TCS SP8 gated STED Super Resolution microscope. Entire cell volumes were captured by acquisition of fine *z*-stacks, 0.24 µm step size for each cell on a 63x objective (NA = 1.4). Images were analyzed utilizing Huygens Essential (Scientific Volume Imaging) and Imaris 7.7.2 (Bitplane A.G.) software.

### Fluorescence in situ hybridization

Fluorescence in situ hybridization was essentially carried out as previously described^32^. Briefly, MDCK II cells were seeded onto glass plates and subsequently infected at an MOI of 5 for 1 h at room temperature, washed three times and further cultivated for 6 h (37 °C, 5% CO_2_). Cells were fixed using 4% PFA and permeabilized overnight (70% EtOH). Segment-specific SC35M DNA probes (Supplementary Table 1) targeting HA (CAL Fluor Red 590), PB2 (Quasar 670) and NP (Quasar 570) vRNAs were purchased from BioSearch Technologies. To ensure specificity, each oligo was compared to the entire SC35M genome (plus and minus strands). Any oligos with 10 base pair similarity to a region other than the desired vRNA segment were excluded. NP protein was visualized using a specific NP antibody (ATCC, H16-L10-4R5; dilution 1 : 1500) and a secondary antibody coupled to AlexaFluor 488 (Jackson ImmunoResearch, 1 : 500). Spectral separation was achieved using a LEICA TCS SP8 gated STED Super Resolution microscope. A sequential scanning program was established based on the manufacturer’s excitation and emission spectra for each fluorophore. The following parameters were used: HyD1 (Quasar 670), range 675–737 nm, excitation wavelength 647 nm (WLL, power 3%), PMT1 (DAPI), range 415–470 nm, excitation wavelength 405 nm (UV laser, power 5%), PMT3 (CAL Fluor Red 590), range 595–650 nm, excitation wavelength 582 nm (WLL, power 10%), HyD2 (Quasar 570), range 550–570 nm, excitation wavelength 545 nm (WLL, power 15%) and PMT1 (488), range 500–539 nm, excitation wavelength 488 nm (WLL, power 5%). Spectral separation was confirmed in each experiment with single color controls (infected cells stained with only a single vRNA FISH probe set or NP antibody). Entire cell volumes were captured by acquisition of fine z-stacks, 0.13 µm step size for each cell on a 63x objective (NA = 1.4) with a zoom of 4. The resultant images had a pixel size of 50 × 50 × 170 nm, which is close to nyquest sampling. The three-dimensional confocal stacks of FISH were background subtracted and deconvolved using Huygens Professional (version 16.05; Scientific Volume Imaging B.V.). Deconvolution was done automatically at 40 iterations per deconvolution to a signal to noise ratio of 20. The resulting images were analyzed with Imaris software (version 8.3.0; Bitplane A.G.). Imaris was used to generate spots for all three FISH probes using the program’s ‘Spots’ feature, and threshold was defined based on 2× the s.d. of the channel mean intensity. To quantify the spots, we first defined the cellular and nuclear spaces for a cell of interest. We used the DAPI channel to define the cell nuclear voxel space with a smoothing of 0.09, and NP protein signal to define the cellular membrane. We then utilized the spots colocalization feature in Imaris MATLAB extensions to identify colocalized spots i.e. spots within 300 nm of each other. The frequency data generated by Imaris was graphed and compared using Prism software (version 6.0, GraphPad).

### Mass spectrometry

NP acetylation sites were detected upon transient expression of NP in HEK293T cells or vRNPs isolated from infected A549 cells. HEK293T cells were transfected with expression plasmids encoding WSN NP-HA (2 µg) and CBP (4 µg). 24 h post transfection, cells were washed and lysed using RIPA buffer (pH = 7.5, 50 mM Tris Base, 150 mM NaCl, 1% NP-40, 1 mM EDTA, 0.25% Sodium-deoxycholate and 0.1% SDS) including 1% Protease Inhibitor Cocktail (Roche). Following sonication and centrifugation, HA-specific NP pulldown using anti-HA agarose (Sigma), was performed overnight at 4 °C. Finally, samples were washed 4 times (RIPA) and subjected to SDS–PAGE. Corresponding gel slices of acetylated NP were excised and digested with trypsin for 20 h. The digested peptides were analyzed by mass spectrometry (LCQ Deca XP Plus, Thermo Finnigan). Data analysis was carried out using Mascot2.2 software (Matrix Science, London, UK; version 2.2). A549 cells were infected with either SC35M or WSN encoding Strep-tagged PB2 at an MOI of 20 and incubated for 20 h at 37 °C and 5% CO_2_. Cells were subsequently washed, using ice-cold PBS and pelleted by centrifugation (1500 r.p.m., 4 °C, 5 min). Lysis was done on ice for 20 min (50 mM TRIS, pH 7.5, 150 mM NaCl, 2 mM MgCl_2_, 2 mM CaCl_2_, 0.7% NP-40, 1 mM DTT, 1% Protease Inhibitor Mix G (Serva) and 50 U per ml DNase I (Thermo Fisher Scientific). Following centrifugation (13,200 r.p.m., 4 °C, 15 min) supernatant was transferred onto Streptavidin Beads (Pierce). Co-immunoprecipitation was performed overnight at 4 °C and precipitates were washed three times in 50 mM TRIS, pH 7.5 containing 150 mM NaCl, 2 mM MgCl_2_, 2 mM CaCl_2_, 0.7% NP-40, 1 mM DTT, 1% Protease Inhibitor Mix G and resuspended in SDS–PAGE sample buffer. Samples were heated in SDS–PAGE loading buffer, reduced with 1 mM DTT for 5 min at 95 °C and alkylated using 5.5 mM iodoacetamide for 30 min at room temperature. Protein mixtures were separated on 4–12% gradient gels. The band corresponding to NP was cut, the proteins were in‐gel digested with either trypsin or elastase and the resulting peptide mixtures were processed on STAGE tips and analyzed by LC-MS/MS^40^. Mass spectrometric (MS) measurements were performed on an LTQ Orbitrap XL mass spectrometer coupled to an Agilent 1200 nanoflow–HPLC. HPLC–column tips (fused silica) with 75 µm inner diameter were self-packed with Reprosil–Pur 120 ODS–3 to a length of 20 cm. Samples were applied directly onto the column without a pre–column. A gradient of A (0.5% acetic acid in water) and B (0.5% acetic acid in 80% acetonitrile in water) with increasing organic proportion was used for peptide separation (loading of sample with 2% B; separation ramp: from 10–30% B within 80 min). The flow rate was 250 nL per min and for sample application 500 nL per min. The mass spectrometer was operated in the data-dependent mode and switched automatically between MS (max. of 1 × 10^6^ ions) and MS/MS. Each MS scan was followed by a maximum of five MS/MS scans in the linear ion trap using normalized collision energy of 35% and a target value of 5000. Parent ions with a charge state from *z* = 1 and unassigned charge states were excluded for fragmentation. The mass range for MS was *m/z* = 370–2000. The resolution was set to 60,000. MS parameters were as follows: spray voltage 2.3 kV; no sheath and auxiliary gas flow; ion-transfer tube temperature 125 °C.

The MS raw data files were uploaded into the MaxQuant software version 1.4.1.2 for peak detection, generation of peak lists of mass error corrected peptides, and for database searches^41^. Full-length UniProt human or dog databases additionally containing all influenza proteins and common contaminants such as keratins and enzymes used for in–gel digestion were used as reference. Carbamidomethylcysteine was set as fixed modification and protein amino–terminal acetylation, oxidation of methionine and lysine acetylation were set as variable modifications. Three missed cleavages were allowed, enzyme specificity was trypsin/P, and the MS/MS tolerance was set to 0.5 Da. The average mass precision of identified peptides was in general less than 1 ppm after recalibration. Peptide lists were further used by MaxQuant to identify and relatively quantify proteins using the following parameters: peptide and protein false discovery rates, based on a forward-reverse database, were set to 0.01, minimum peptide length was set to 6, minimum number of peptides for identification and quantitation of proteins was set to one which must be unique, minimum ratio count was set to two, and identified proteins were requantified. The ‘match-between-run’ option (2 min) was used.

### Polymerase reconstitution assay

Polymerase reconstitution was performed as previously described^42^. Briefly, pCAGGs plasmids encoding PB2, PB1, PA (each 45 ng) and NP (150 ng) were co-transfected with the firefly luciferase-encoding viral minigenome construct pPolI-FFLuc-RT (25 ng) and a plasmid (pRL-SV40, 20 ng) coding for *Renilla* luciferase in HEK293T cells. 20 h post transfection, cells were lysed and firefly and *Renilla* luciferase activities were measured using the Dual-Luciferase® Reporter assay system (Promega). For co-immunoprecipitation, pCAGGs plasmids encoding PB2, PB1, PA (each 600 ng) and NP (1500 ng) were co-transfected with the firefly luciferase-encoding viral minigenome construct pPolI-FFLuc-RT (200 ng) in HEK293T cells. 24 h post transfection, cells were washed, using ice-cold PBS and pelleted by centrifugation (1500 r.p.m., 4 °C, 5 min). Lysis was done on ice for 20 min (50 mM TRIS, pH 7.5, 150 mM NaCl, 2 mM MgCl_2_, 2 mM CaCl_2_, 0.7% NP-40, 1 mM DTT, 1% Protease Inhibitor Mix G (Serva) and 50 U per ml DNase I (Thermo Fisher Scientific)). Following centrifugation (13.200 r.p.m., 4 °C, 15 min) supernatant was transferred onto 1 µl NP-specific antibodies (ATCC: H16-L10-4R5) and binding was performed overnight at 4 °C. Protein A Sepharose (GE Healthcare) was subsequently used to immunoprecipitate NP (1 h, 4 °C). Afterwards, samples were washed 3 times in 50 mM TRIS, pH 7.5 containing 150 mM NaCl, 2 mM MgCl_2_, 2 mM CaCl_2_, 0.7% NP-40, 1 mM DTT, 1% Protease Inhibitor Mix G and resuspended in SDS–PAGE sample buffer.

### Primer extension analysis

To determine viral transcript levels HEK293T cells were transfected with pCAGGs plasmids encoding PB2, PB1, PA (each 250 ng) and NP (1000 ng). 20 h post transfection, media was exchanged and cells were further incubated with infection media containing cycloheximide (100 µg per mL) for 1 h at 37 °C and 5% CO_2._ Infection was subsequently carried out at an MOI of 5 for 6 h in infection media (cycloheximide: 100 µg per mL). Afterwards, cells were collected in Trizol and RNA was purified according to the manufacturer’s protocol (Zymo Research). Primer extension analysis was performed using specific primers for the PB2 segment (mRNA, cRNA and vRNA) and cellular ribosomal RNA (5sRNA) as previously described^9^. Uncropped scans can be found in Supplementary Fig. 8.

### Transmission electron microscopy

Transmission electron studies were essentially carried out as described before^43^. MDCK II cells were cultivated at 37 °C and 5% CO_2_. To synchronize infection, cells were infected on ice at an MOI of 10 for 1 h. Subsequent temperature shift to 37 °C (10 min) ensured virus internalization and remaining viral particles were removed by three times washing with PBS. Afterwards, cells were cultured in infection media at 37 °C and 5% CO_2_. At 10 h post infection, cells were fixed (0.1 M PHEM, 4% PFA, 0.1% Glutaraldehyde) for 1 h at room temperature. Fixative solution was removed via centrifugation and replaced by 500 µl PHEM (0.1 M) containing 4% PFA. Cells were post-fixed with 1 % osmium tetroxide, dehydrated and embedded in epoxy resins. Ultrathin sections were analyzed using a JEM1400 transmission electron microscope (TEM).

### Virus-like particle assay

Virus-like particles harboring eight segment were generated by transfecting HEK293T cells with pHW2000 plasmids encoding SC35M HA, NA, PA, PB2, M and NS together with the respective pHW2000_NP, pHW400_PB1_eGFP and expression plasmid pCAGGs SC35M PB1 (each 500 ng). As negative control, M1 protein was omitted. Supernatant was collected 48 h post transfection and transferred onto MDCK II cells together with SC35M (MOI 5). GFP expression was measured 12–16 h post transfection on HEK293T cells and infection of MDCK II cells using an Axio Observer.Z1 (Zeiss) microscope.

### Quantitative RT–PCR analysis of incorporated vRNAs

MDCK II cells were infected at an MOI of 0.001 and supernatant was collected between 16–24 h post infection. Viral particles were pelleted by ultracentrifugation (40% sucrose, 90,000 *g* for 1.5 h at 4 °C) and resuspended in PBS. Total RNA was extracted from the viral preparations containing the same amount of infectious particles (PFU). Using segment-specific primers (Supplementary Table 2), the relative number of vRNA using SYBR green real time PCR was quantified as previously described^9^. Relative virion vRNA levels were determined as described^9^ and compared between mutant and wt virus. Results are presented from at least three independent virus stocks. Genome levels obtained with nuclear or cytoplasmic fractions were normalized to GAPDH RNA levels detected using forward primer GF (5′-GGAGCGAGATCCCTCCAAAAT-3′) and reverse primer GR (5′-GGCTGTTGTCATACTTCTCATGG-3′).

### Statistics

If not indicated otherwise, Student’s *t*-test was used for two-group comparisons. The *p-value < 0.05, **p-value < 0.01, and ***p-value < 0.001 were considered significant. Unless otherwise noted, *error bars* indicate the mean and s.d. of at least three independent experiments.

### Data availability

The authors declare that all data supporting the findings of this study are available within the article and its Supplementary Information files, or are available from the authors upon request.

## Electronic supplementary material

Supplementary Information
