# Supplementary material for: Role of influenza A virus NP acetylation on viral growth and replication
Source: Nat Commun. 2017 Nov 2;8:1259. doi: 10.1038/s41467-017-01112-3 (PMC5668263; doi:10.1038/s41467-017-01112-3)
Supplement: Supplementary file 1 — Supplementary Information [file 41467_2017_1112_MOESM1_ESM.pdf]

a

NP protein WSN (H1N1)	Conserv. [%] (n=27,675)
K4	0.1
K7	100
K31	21.4
K48	98.4
K77	79.3
K87	99.5
K90	99.9
K91	100
K103	95.7
K113	100
K184	100
K198	99.9
K227	100
K229	100
K273	100
K325	100
K351	52.1
K357	52.8
K470	94.9

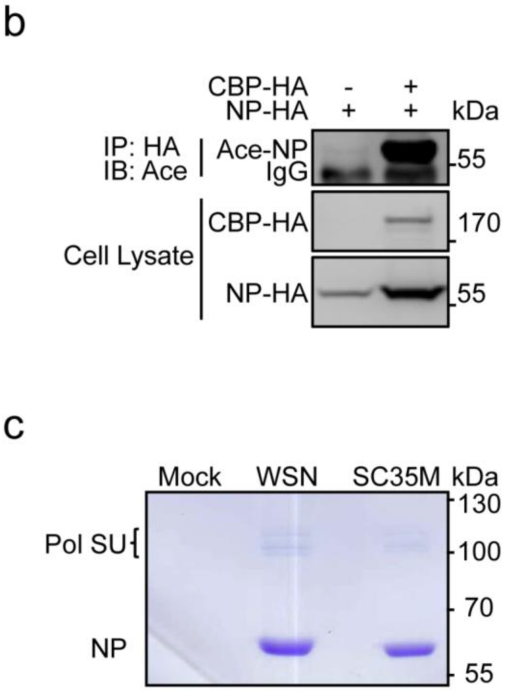

Supplementary Figure 1. Acetylation of highly conserved lysine residues of influenza A virus (IAV) NP.

(**a**) IAV NP encodes about 19 lysine (K) residues, most of them being highly conserved. WSN NP sequence (ABF21292.1) was used as consensus sequence. A total of 27,675 sequences of NP protein of influenza A isolates were downloaded from National Center for Biotechnology Information database and aligned using MEGA6.

(**b**) HEK293T cells were transfected with C-terminal HA-tagged WSN NP (NP-HA) in the presence or absence of the HA-tagged CREB-binding protein (CBP-HA). 24 h.p.t., cells were lysed and NP was immunoprecipitated (IP), followed by SDS-PAGE

and visualization of acetylated NP using a pan-acetylation antibody. NP and CBP levels were determined using HA- specific antibodies.

(**c**) A549 cells were infected for 20 h (MOI: 20), using recombinant WSN (WSN-StrepPB2) or SC35M (SC35M-StrepPB2) encoding Strep-tagged PB2. Following immunoprecipitation using Streptavidin, the native vRNP complex comprising the polymerase subunits PB2, PB1 and PA (Pol Sub), together with NP was separated by SDS-PAGE and stained by Coomassie Blue.

a

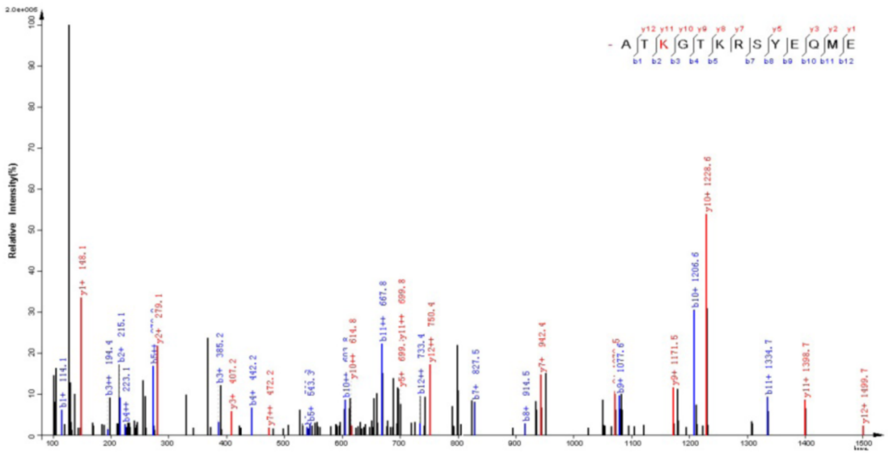
A T K G T K R S Y E Q M E
Relative Intensity(%)

K4

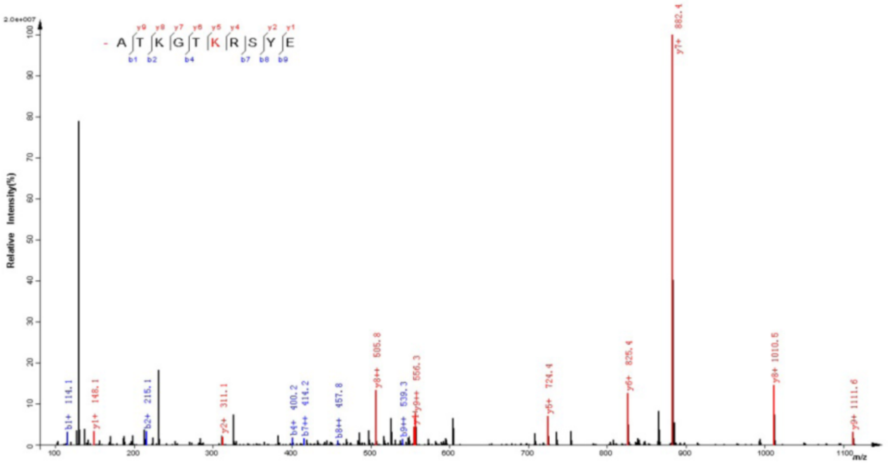
A T K G T K R S Y E
Relative Intensity(%)
m/z

K7

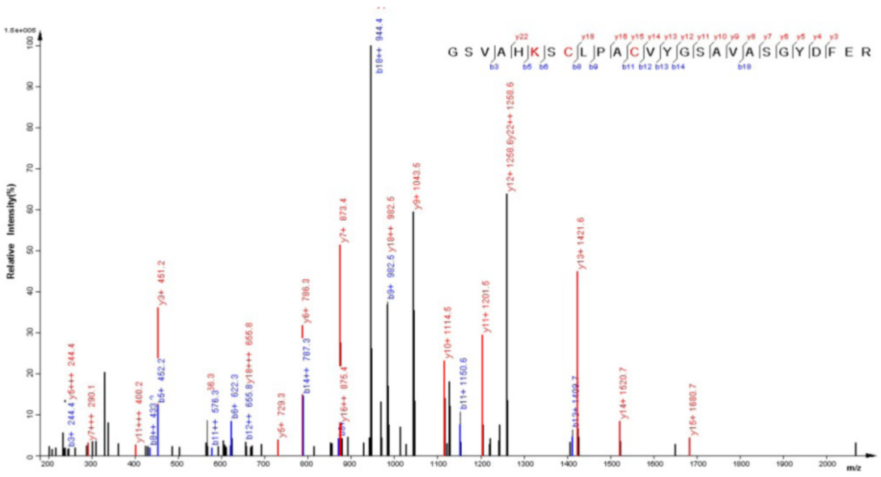
G S V A H K S C L P A C V Y G S A V A S G Y D F E R
Relative Intensity(%)
m/z

K273

b

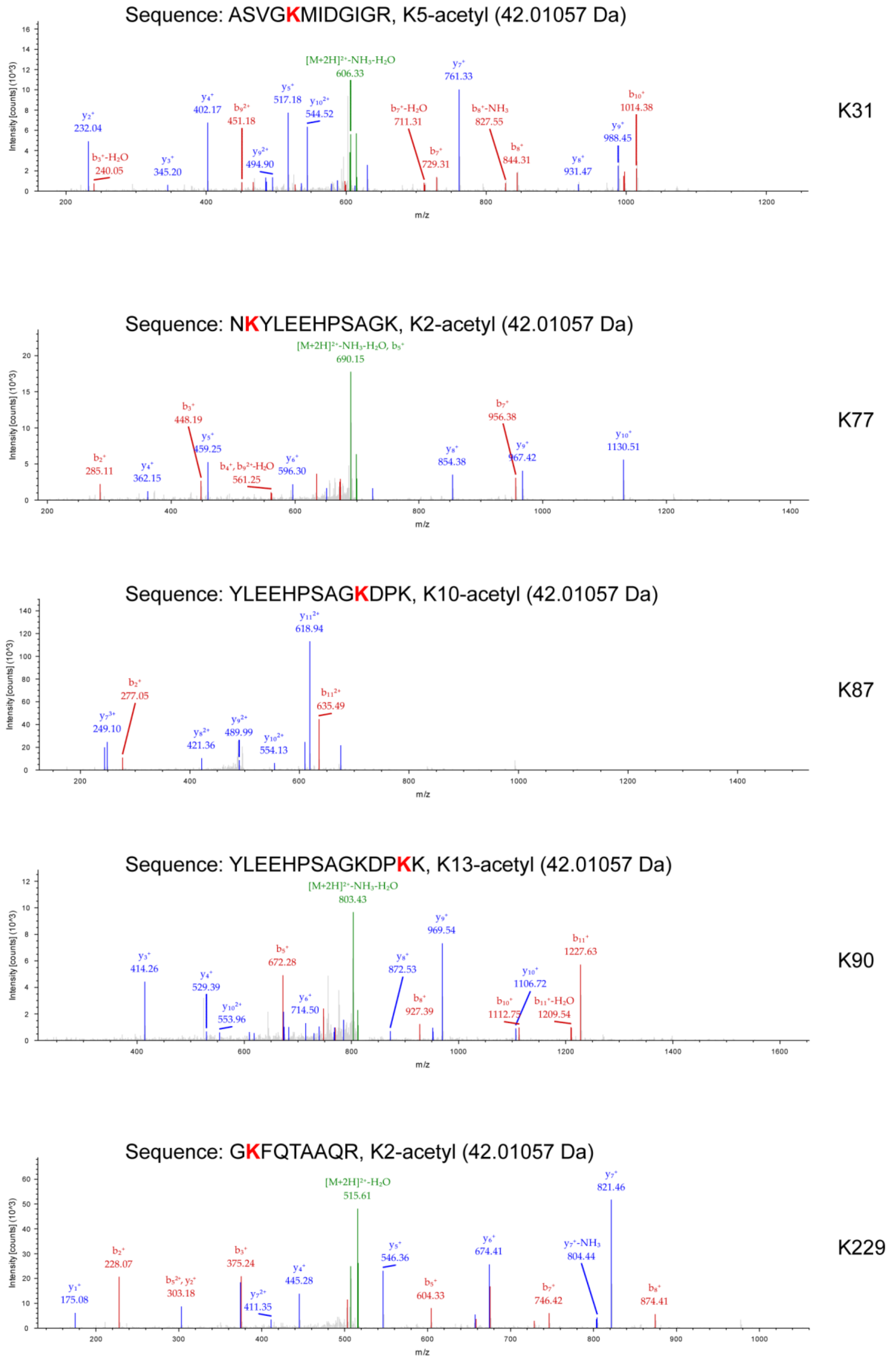
Sequence: ASVGKMIDGIGR, K5-acetyl (42.01057 Da)
K31
[M+2H]²⁺-NH₃-H₂O 606.33
y₇⁺ 761.33
y₂⁺ 232.04
b₃⁺-H₂O 240.05
y₃⁺ 345.20
y₄⁺ 402.17
b₉²⁺ 451.18
y₉²⁺ 494.90
y₅⁺ 517.18
y₁₀²⁺ 544.52
b₇⁺-H₂O 711.31
b₇⁺ 729.31
b₈⁺-NH₃ 827.55
b₈⁺ 844.31
y₈⁺ 931.47
y₉⁺ 988.45
b₁₀⁺ 1014.38
Intensity [counts] (10^3)
m/z
Sequence: NKYLEEHPSAGK, K2-acetyl (42.01057 Da)
K77
[M+2H]²⁺-NH₃-H₂O, b₅⁺ 690.15
b₂⁺ 285.11
y₄⁺ 362.15
b₃⁺ 448.19
y₃⁺ 459.25
b₄⁺, b₉²⁺-H₂O 561.25
y₆⁺ 596.30
y₈⁺ 854.38
b₇⁺ 956.38
y₉⁺ 967.42
y₁₀⁺ 1130.51
Intensity [counts] (10^3)
m/z
Sequence: YLEEHPSAGKDPK, K10-acetyl (42.01057 Da)
K87
y₁₁²⁺ 618.94
y₇³⁺ 249.10
b₂⁺ 277.05
y₈²⁺ 421.36
y₉²⁺ 489.99
y₁₀²⁺ 554.13
b₁₁²⁺ 635.49
Intensity [counts] (10^3)
m/z
Sequence: YLEEHPSAGKDPKK, K13-acetyl (42.01057 Da)
K90
[M+2H]²⁺-NH₃-H₂O 803.43
y₃⁺ 414.26
y₄⁺ 529.39
y₁₀²⁺ 553.96
b₅⁺ 672.28
y₆⁺ 714.50
y₈⁺ 872.53
b₈⁺ 927.39
y₉⁺ 969.54
b₁₀⁺ 1112.75
y₁₀⁺ 1106.72
b₁₁⁺-H₂O 1209.54
b₁₁⁺ 1227.63
Intensity [counts] (10^3)
m/z
Sequence: GKFQTAAQR, K2-acetyl (42.01057 Da)
K229
[M+2H]²⁺-H₂O 515.61
y₁⁺ 175.08
b₂⁺ 228.07
b₅²⁺, y₂⁺ 303.18
b₃⁺ 375.24
y₇²⁺ 411.35
y₄⁺ 445.28
y₅⁺ 546.36
b₅⁺ 604.33
y₆⁺ 674.41
b₇⁺ 746.42
y₇⁺-NH₃ 804.44
y₇⁺ 821.46
b₈⁺ 874.41
Intensity [counts] (10^3)
m/z

C

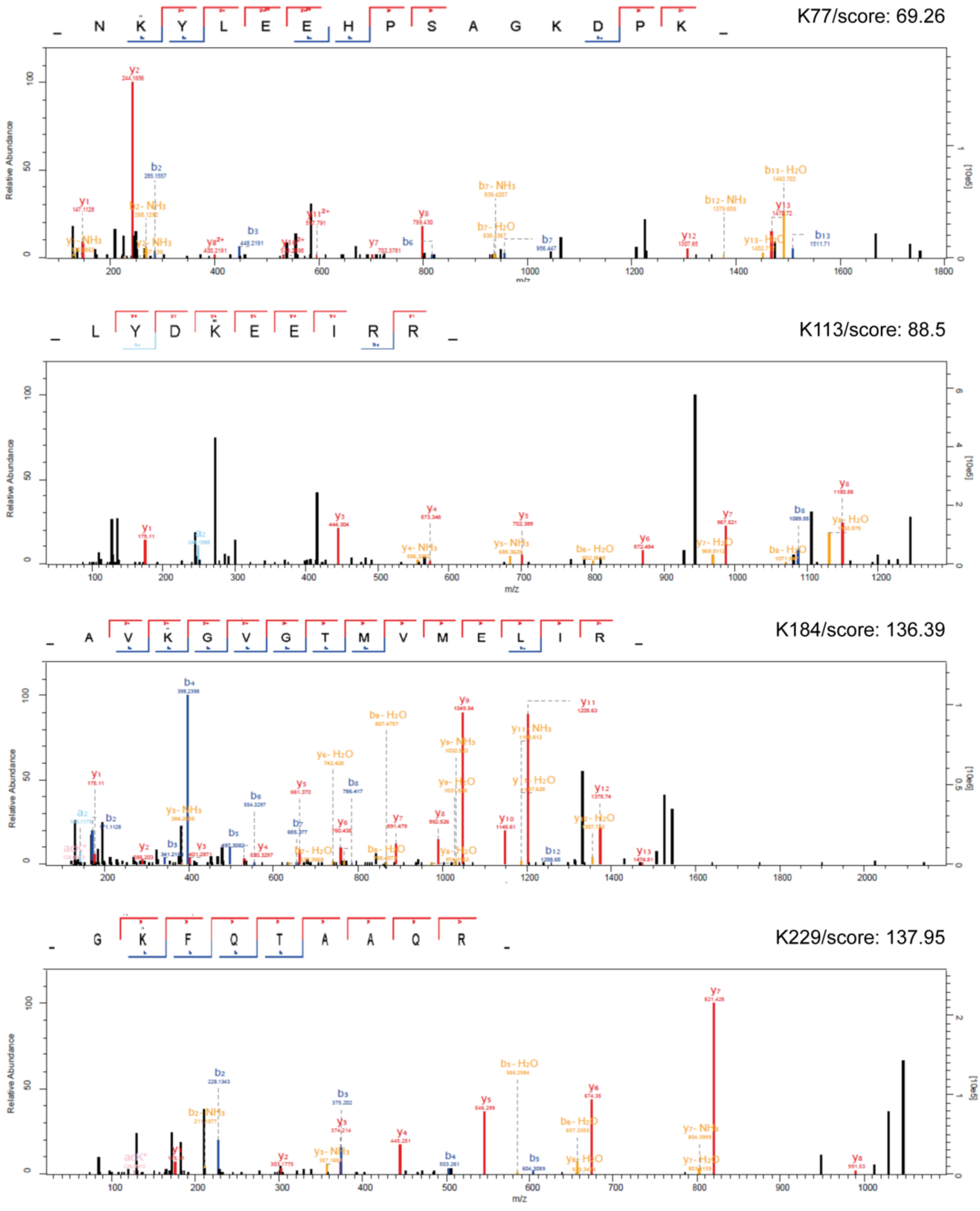

K77/score: 69.26
N K Y L E E H P S A G K D P K
Relative Abundance
m/z
[10e5]
K113/score: 88.5
L Y D K E E I R R
Relative Abundance
m/z
[10e5]
K184/score: 136.39
A V K G V G T M V M E L I R
Relative Abundance
[10e6]
K229/score: 137.95
G K F Q T A A Q R
Relative Abundance
m/z
[10e5]

Supplementary Figure 2. NP acetylation sites identified via Mass Spectrometry

(**a, b**) WSN NP acetylation sites identified upon transfection of HEK293T cells in presence of CREB-binding protein (CBP). Peptide sequences from tandem mass spectrometry containing the acetylation sites K4, K7, K31, K77, K87, K90, K229 and K273 are shown.

(**c**) NP acetylation sites identified using Strep-tagged-purified vRNPs of WSN and SC35M. Peptide sequences from tandem mass spectrometry containing the acetylation sites K77, K113, K184 and K229 are shown.

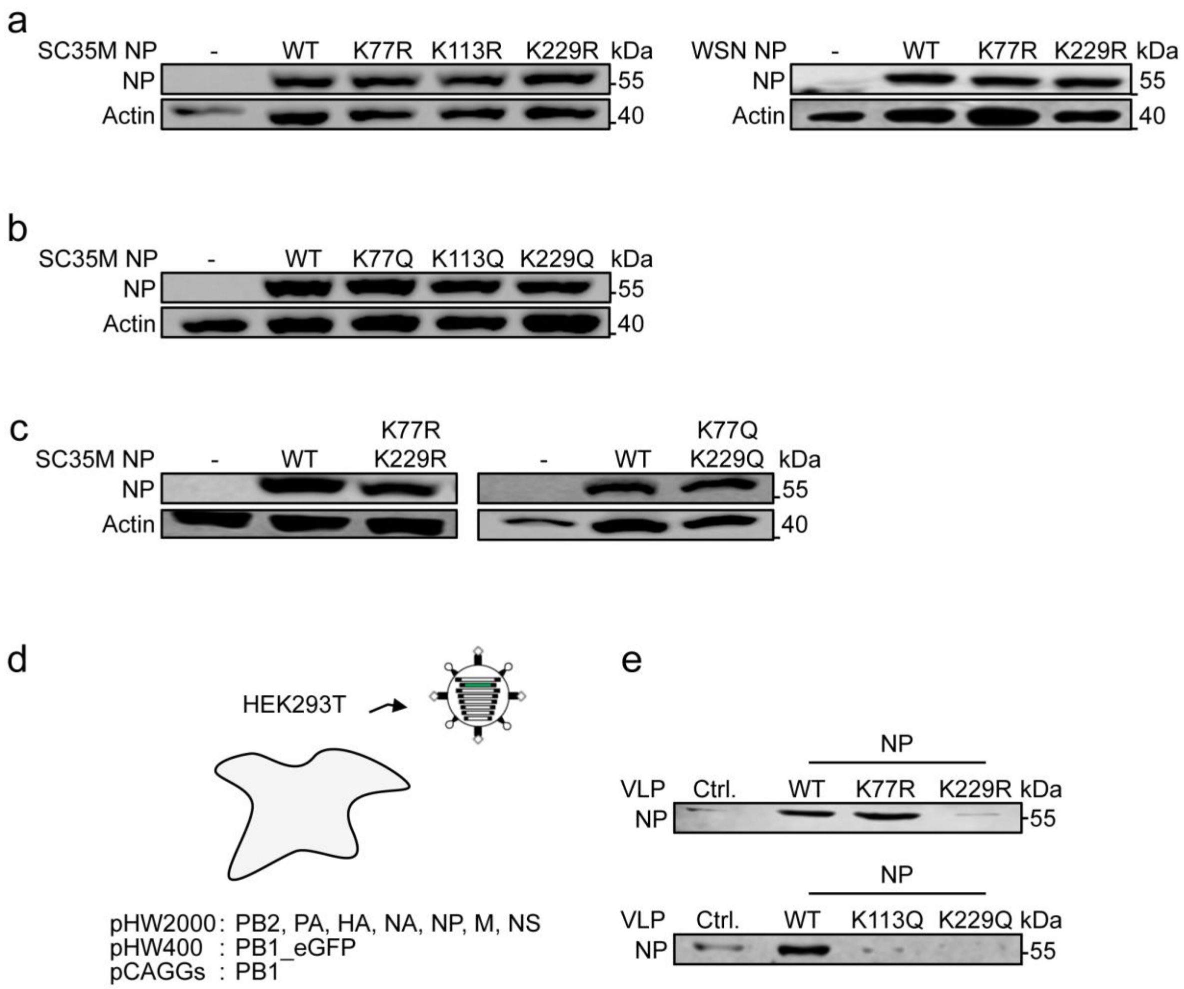

Supplementary Figure 3. NP acetylation mimics do not affect protein stability but impair genome incorporation into budding viral particles.

(**a, b, c**) HEK293T cells were transiently transfected with pCAGGs expression plasmids encoding the indicated NP mutants. 20 h.p.t., total cell lysate was used to determine NP expression. Actin serves as cellular loading control.

(**d**, **e**) HEK293T cells were transiently transfected as depicted (**d**). At 48 h.p.t., supernatant containing virus-like particles (VLPs) was collected and analyzed by SDS-PAGE. Total NP content within the HEK293T supernatant is shown (**e**).

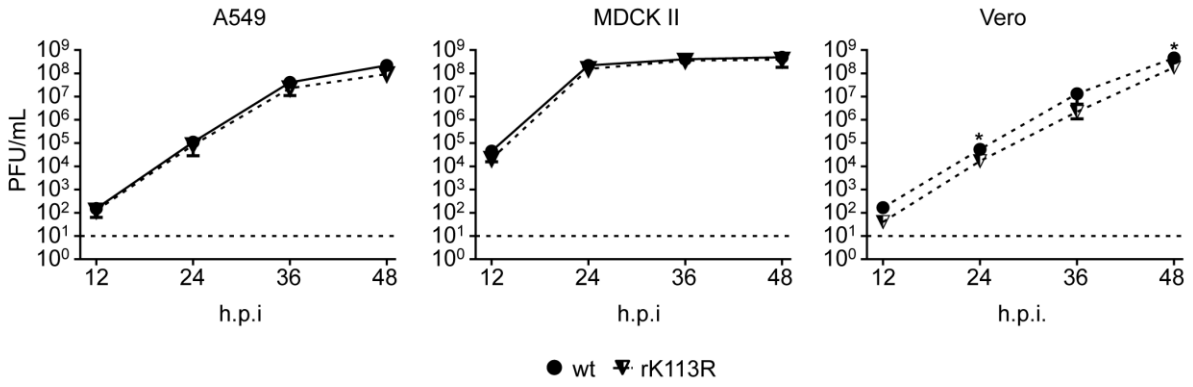

Supplementary Figure 4. NP mutant virus rK113R reveals no growth deficiency. A549, MDCK II and Vero cells were infected with the indicated viruses at an MOI of 0.001 and viral growth was monitored 12, 24, 36 and 48 h.p.i. by plaque assay. Dashed lines represent the detection limit. *p<0.05. Error bars indicate the mean and s.e.m. of at least three independent experiments. Student's t test was used for two-group comparisons.

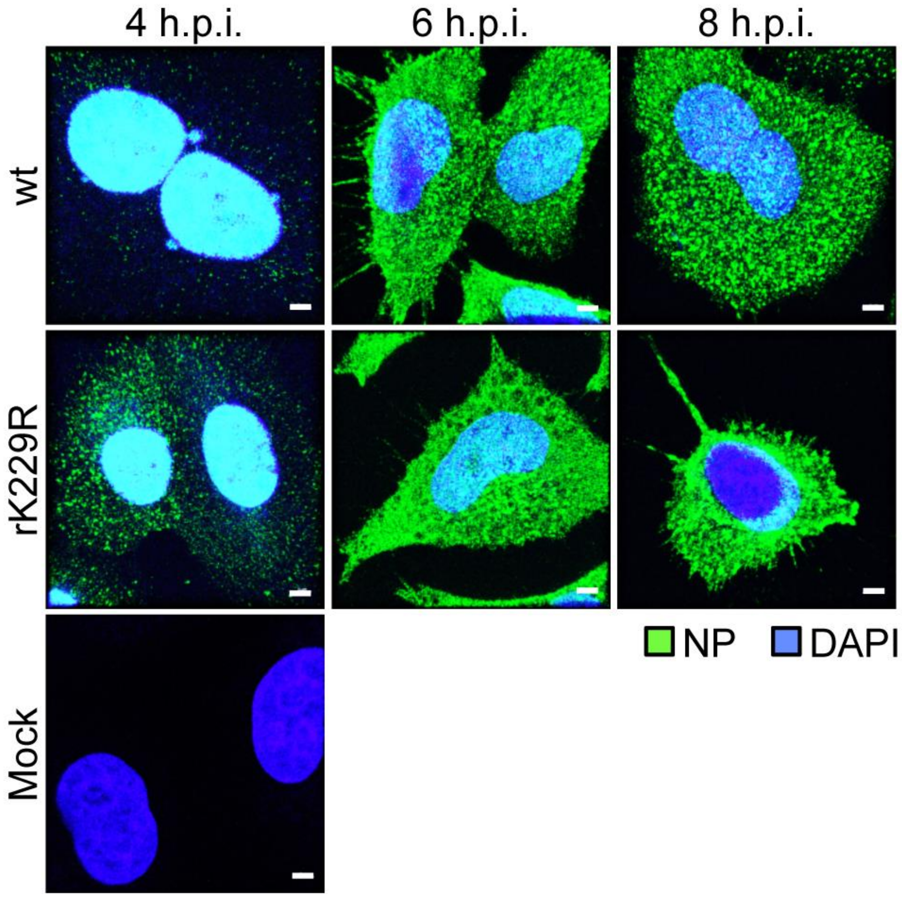

Supplementary Figure 5. NP mutant virus rK229R does not display altered NP localization in human A549 cells.

Subcellular localization of NP in human A549 cells infected with the indicated viruses at an MOI of 5 after 4, 6 and 8 h.p.i. DAPI staining defines the host cell nucleus. Scale bar = 3 µm.

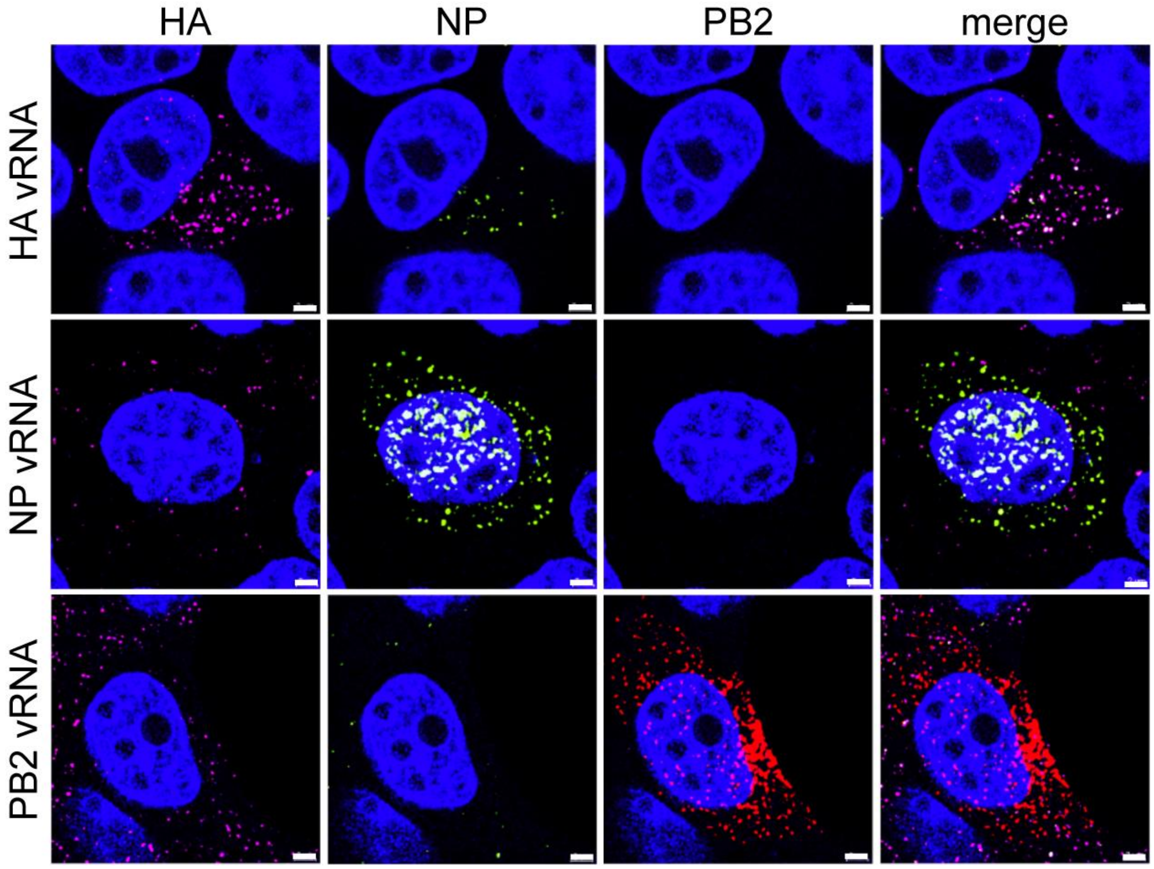

Supplementary Figure 6. Single color controls to visualize three distinct fluorophores.

MDCK II cells infected with SC35M wt at an MOI of 5 for 6 h.p.i. were probed with a single fluorescently-labeled FISH probe and visualized using the three-color imaging parameters. Note, despite minimal bleed-through for HA and NP vRNA probes, fluorescence of each probe was mostly specific for the respective channel. Marginal inaccuracy due to tight excitation spectra was observed for each virus and thus considered negligible regarding the final analysis. Scale bar = 2 µm.

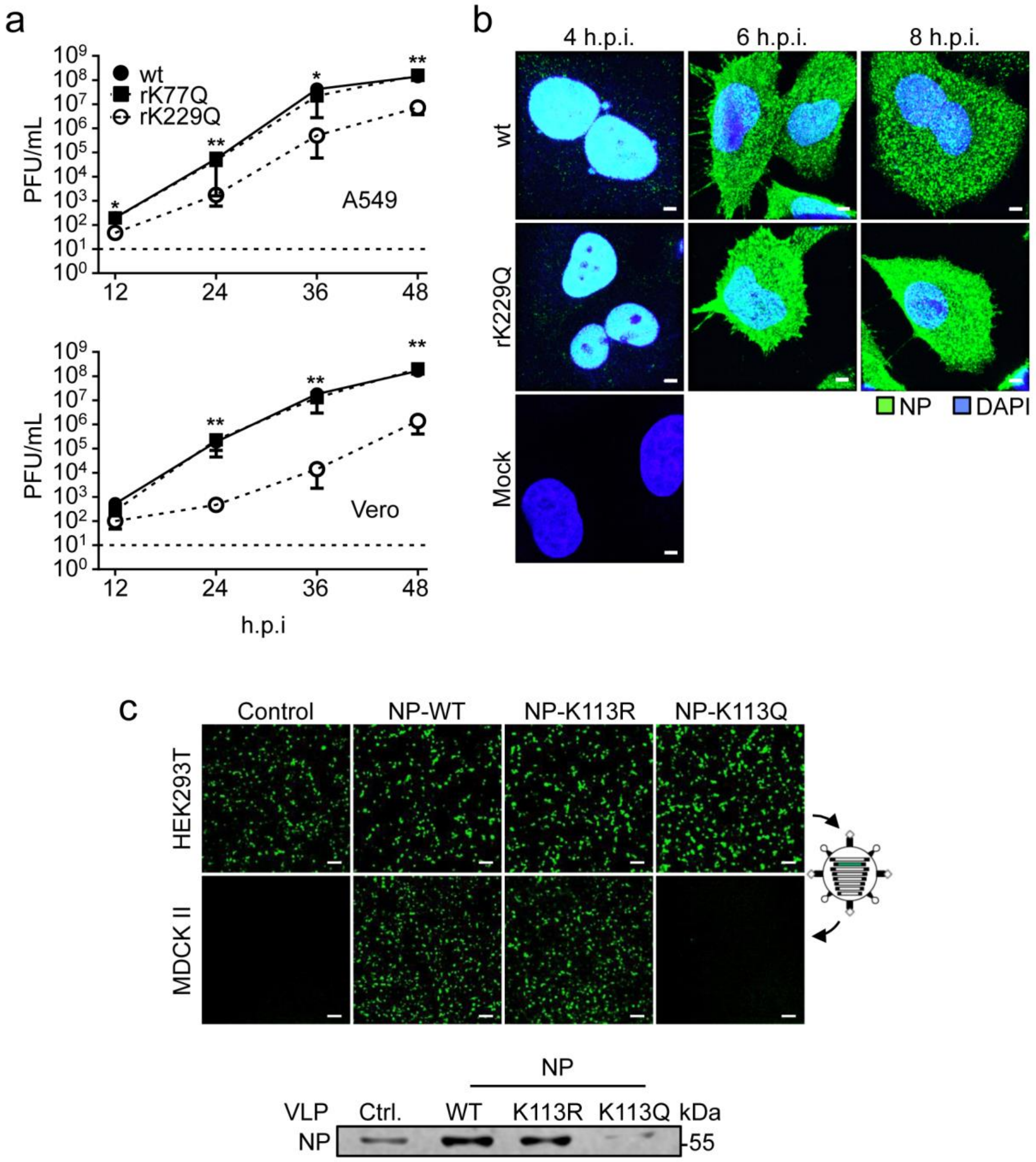

Supplementary Figure 7. Mimicking constant acetylation at K229 attenuates viral growth.

(**a**) A549 and Vero cells were infected with the indicated viruses at an MOI of 0.001 and viral growth was monitored 12, 24, 36 and 48 h.p.i. by plaque assay. Dashed lines represent the detection limit. *p< 0.05 **p< 0.01. Student‘s t test was used for

two-group comparisons. Error bars indicate the mean and s.e.m. of at least three independent experiments.

(**b**) Subcellular localization of NP in human A549 cells infected with the indicated viruses at an MOI of 5 after 4, 6 and 8 h.p.i. Scale bar = 3 µm.

(**c**) VLPs were generated in HEK293T cells in the presence of a GFP-encoding reporter segment with the remaining 7 genome segments to determine the genome incorporation efficiency mediated by wt or mutant SC35M NP protein. VLPs released into the cell supernatant were used to infect MDCK II cells, which were subsequently superinfected with SC35M. Scale bar = 100 µm. Supernatant containing virus-like particles (VLPs) was collected and analysed by SDS-PAGE. Total NP content within HEK293T supernatant is shown.

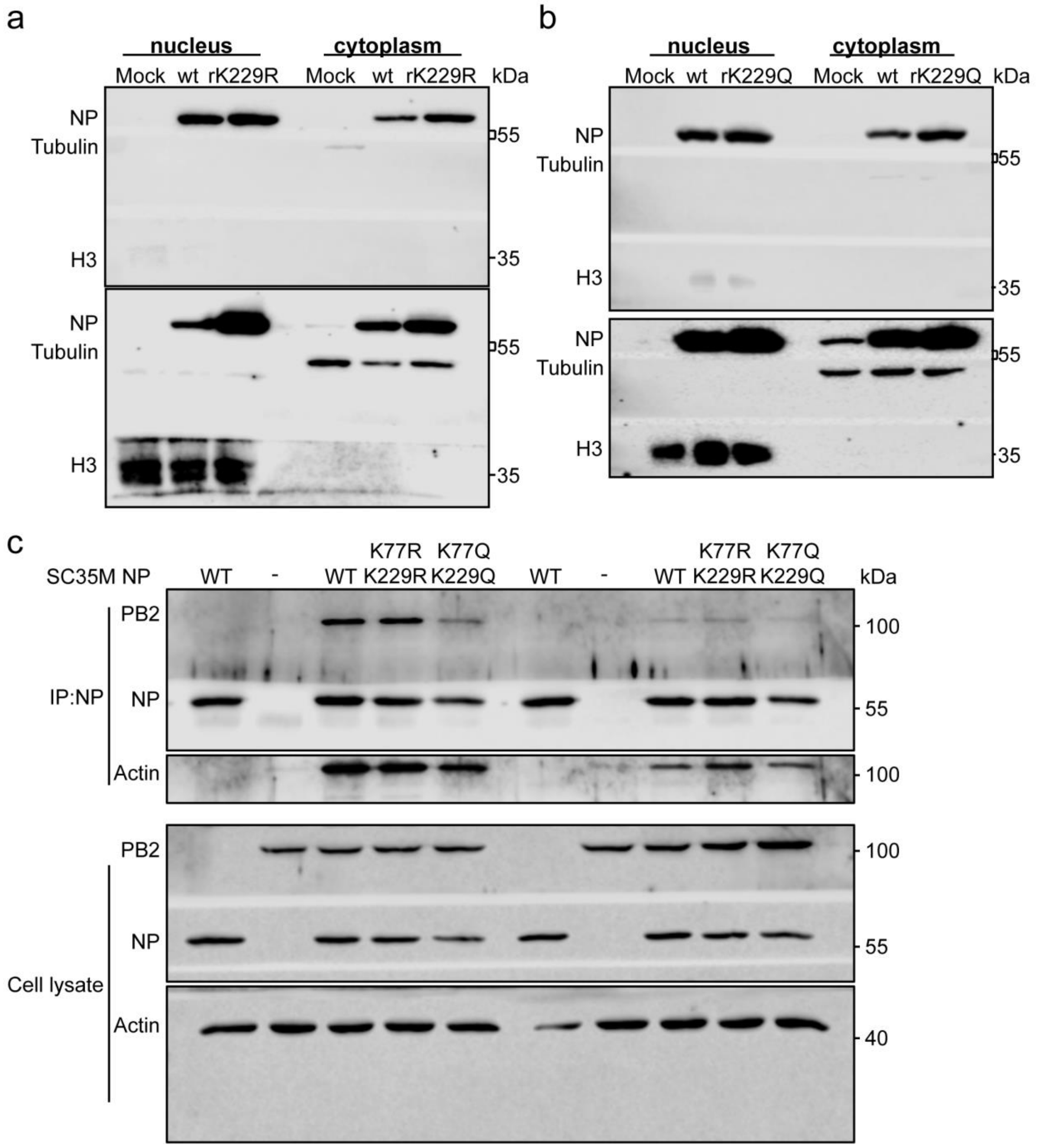
a
nucleus
cytoplasm
Mock wt rK229R Mock wt rK229R kDa
NP
Tubulin
H3
55
35
b
nucleus
cytoplasm
Mock wt rK229Q Mock wt rK229Q kDa
NP
Tubulin
H3
55
35
c
SC35M NP WT - WT K77R K229R K77Q K229Q WT - WT K77R K229R K77Q K229Q kDa
IP:NP
PB2
NP
Actin
Cell lysate
PB2
NP
Actin
100
55
100
100
55
40

d

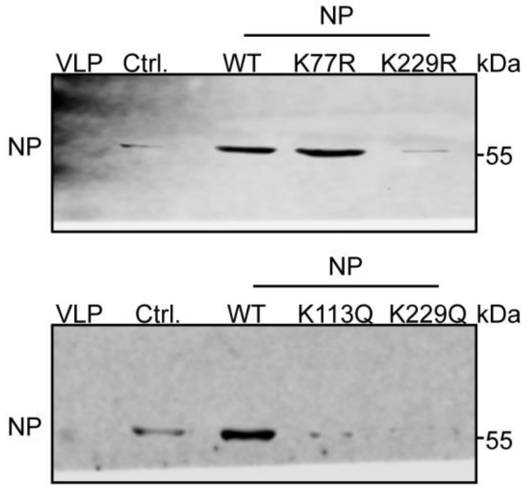

e

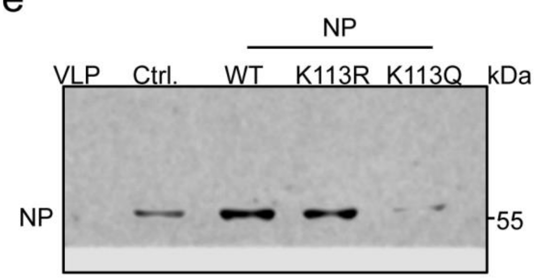

f

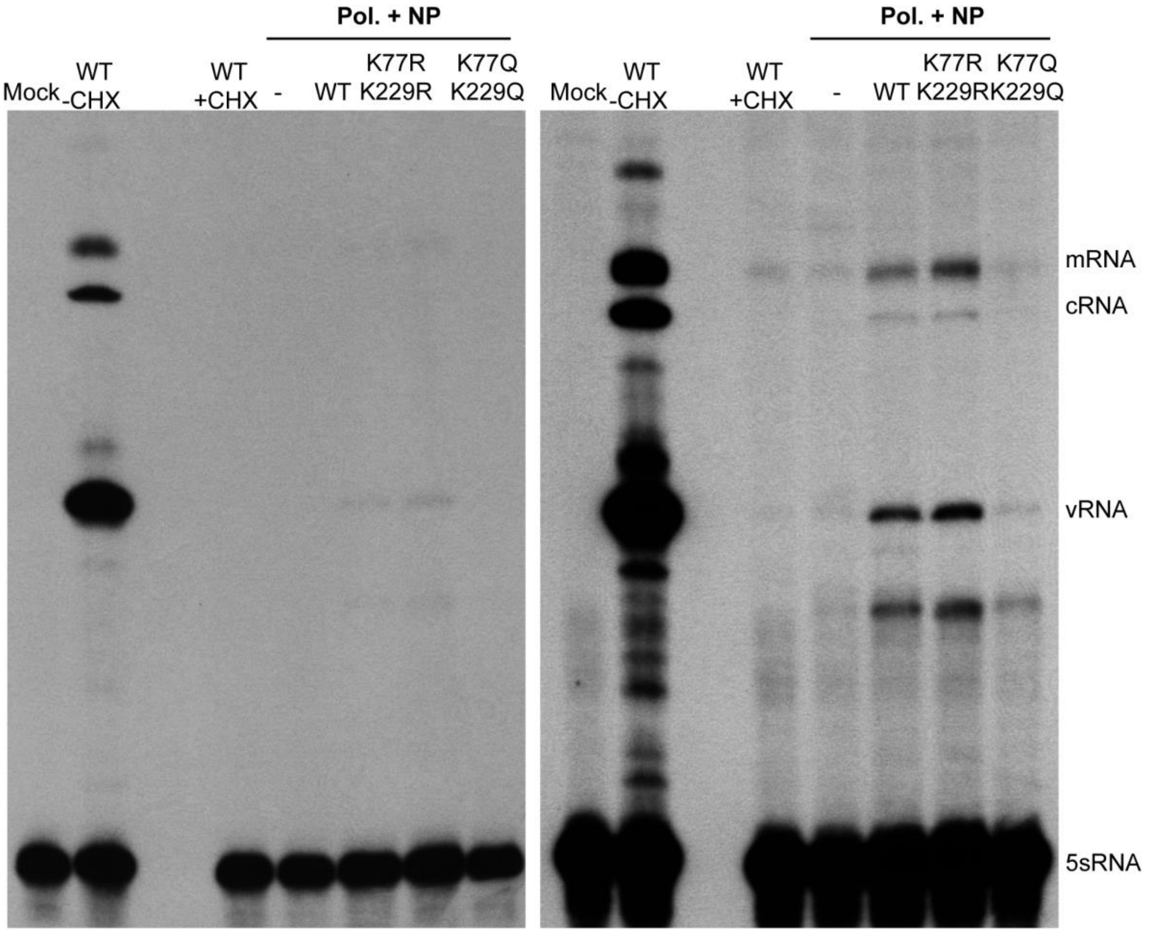

Supplementary Figure 8. Uncropped scans.

(**a**) Uncropped scans of Figure 2b. The membrane was cut and the stained against NP, ß-Tubulin and histone H3 as indicated.

(**b**) Uncropped scans of Figure 4d. The membrane was cut and stained
against NP, ß-Tubulin and histone H3 as indicated.

(**c**) Uncropped scans of Figure 5c (left) and Figure 5d (right). The membrane was cut and stained against PB2, NP and actin as indicated.

(**d**) Uncropped scans of Supplementary Figure 3e. The membrane was cut and stained against NP.

(**e**) Uncropped scans of Supplementary Figure 7c. The membrane was cut and stained against NP.

(**f**) Uncropped scans of Figure 5e.

Supplementary Table 1. FISH probe sequences.

#	FISH probes: PB2 (5‘-3‘)	#	FISH probes: HA (5‘-3‘)	#	FISH probes: NP (5‘-3‘)
**1.**	ttcggatggccatcaattag	**1.**	gcggtgcactatttgtatat	**1.**	gagtgacatccacatcatgg
**2.**	tacttactgacagccagaca	**2.**	tagccattgcaatgggattg	**2.**	gcagggtagataatcactca
**3.**	gaaacggaagcgggactcta	**3.**	atcttatggtttagcttcgg	**3.**	gcatctgttgggagaatagc
**4.**	tgtgctaattgggcaaggag	**4.**	cacttatgaccatacccaat	**4.**	ccagaatgccactgaaatca
**5.**	ccagcgttaagcatcaatga	**5.**	attccataagtgtgacgacc	**5.**	gagcagatggaaactggtgg
**6.**	gagaggattcctcattttgg	**6.**	gatgggactggatgttttga	**6.**	ctcagcgactatgaagggag
**7.**	ctcggaaaagatgcaggtgc	**7.**	gcacacaatagatcttgcgg	**7.**	acagatgtgcactgaactca
**8.**	caacaaggccacaaagaggc	**8.**	tgttggtggccatggaaaat	**8.**	ttggtggaatcgggagattc
**9.**	aagaggcaattctccagttt	**9.**	ggacacgagactcaatgact	**9.**	ttctctcggcatttgatgag
**10.**	gggtcaggaatgaggatact	**10.**	gcagtttgagctgatagaca	**10.**	aagaagacagggggtccaat
**11.**	ccaccggaacaaagtaggat	**11.**	aagcacccaatctgcaatag	**11.**	taattggaagagcatcccag
**12.**	atgcgagatgtgcttggaac	**12.**	agaaggaactgcagctgact	**12.**	ccaacctgaatgatgctaca
**13.**	cagaggtcaatacagcggat	**13.**	ggtttcaggcatcaaaatgc	**13.**	ctcacccatttgatgatctg
**14.**	atggaattcgagccatttca	**14.**	agagaatggatgggagggtc	**14.**	tggagaagacgcaactgctg
**15.**	acaggatcccacaatgttat	**15.**	ctttttggagcaattgctgg	**15.**	ctctctgatgcaaggttcaa
**16.**	tggatcatcaggaactggga	**16.**	tgtcccagagaatccaaaga	**16.**	gcagtgaaaggggttggaac
**17.**	cacaagggacggagaagcta	**17.**	tccttttggctacaggaatg	**17.**	aggcgaaaatgggcggagaa
**18.**	ggaatgtactactatcaccc	**18.**	ccgatatgtcaaacagccaa	**18.**	cctgctccaaaacagtcaag
**19.**	ccgttttttaagagttcggg	**19.**	ctagaacggtggggaaatgc	**19.**	ttgagagagagggatactcc
**20.**	gagtcagcaagatgggagta	**20.**	tctgccattccaaaacatca	**20.**	tcaactggtatggatggcat
**21.**	agtactgagatgtcgctgag	**21.**	aggggattgctttcatagtg	**21.**	cccaaagaaaatccagcaca
**22.**	tgtgatgggaatgatcggga	**22.**	tgatgtgcctttagactcta	**22.**	atcagaggagcaagagtggt
**23.**	aaaactggggaattgagccc	**23.**	aggagaatcactaggagtcc	**23.**	agatctgagagtgtcaagct
**24.**	ggcatttccaaaaggatgca	**24.**	cctaatagggcaagtttctt	**24.**	agtactcttgaattgaggcc
**25.**	ggtgacctgaactttgtcaa	**25.**	accttcaatggagcattcat	**25.**	cagaggagttcagattgctt
**26.**	agcaataattgtggccatgg	**26.**	tgatcccaatgacacagtga	**26.**	agagagcatctgcaggacaa
**27.**	gcaaccaggagattgattca	**27.**	tcaatttccattggctactc	**27.**	cagaagcggagggaacacaa
**28.**	ttgggagaagagcaacagct	**28.**	ggccacaagtgaatggacaa	**28.**	agcagatattgggccataag
**29.**	aagaagtgcttaccggcaac	**29.**	accagactctatggaagtgg	**29.**	gagagagcaaccattatggc
**30.**	cggatcgtcagtcaagagag	**30.**	attcaccattctggatcgac	**30.**	ttctcagtgcagagaaacct
**31.**	attagctcatctttcagctt	**31.**	aaccagctctgatagtttgg	**31.**	cgagctctcggacgaaaagg
**32.**	tggatatatgcaaggcagca	**32.**	cgaattcagacaatgcggca	**32.**	tgaagatgtgtctttccagg
**33.**	ctgaggcagaatccaacaga	**33.**	gcggaaatgaagtggttgct	**33.**	ggtcttatttcttcggagac
**34.**	cagcacacagattggtggaa	**34.**	agaagatcaggttcttcctt		
**35.**	caacagtatcagcagatcca	**35.**	ccaatggaacaacaagtgcc		
**36.**	agcttaattattgctgccag	**36.**	gtcaatgggtttcacctata		
**37.**	ccaggaggggaagtgagaaa	**37.**	ggtcaggaggagtcgataaa		
**38.**	ttgcatttgacccaaggaac	**38.**	agaatcactgaggcagatcc		
**39.**	tggtccgcaaaacaaggttc	**39.**	agggaacgatgtgtgctatc		
**40.**	ggattgtaaaattgctccct	**40.**	ccaatgtgatcaattcctgg		
**41.**	ggggccagaatactaacatc	**41.**	taggaaccctaataggacct		
**42.**	catggaagttgttttcccaa	**42.**	caacagacctgggacaatgt		
**43.**	cagaccttagtgccaaagag	**43.**	atctgcacccaagggaaaag		

#	FISH probes: PB2 (5‘-3‘)	#	FISH probes: HA (5‘-3‘)
**44.**	gttgacataaaccctggaca	**44.**	gtggaaaccgcgaatatcgg
**45.**	ggaataggaatgggccaaca	**45.**	gaagtagtgaatgccacgga
**46.**	gcaaaacaaatgacgccggc	**46.**	aacacgctaacagagagggg
**47.**	ggatgaagtggatgatggca	**47.**	atatgtcttggacaccatgc
**48.**	gaagacaggagaagaacccc	**48.**	ctgtgtgctgattgaagcta

Supplementary Table 2. RT-qPCR Primer.

Name	Sequence
RT-qPCR PB2 segment-specific primer forward	gcacaggatgtaatcatggaagt
RT-qPCR PB2 segment-specific primer reverse	accaattctctttctagcatgtatgc
RT-qPCR HA segment-specific primer forward	gctttttggagcaattgctgg
RT-qPCR HA segment-specific primer reverse	gtgcttttgtagtcagctgca
RT-qPCR NP segment-specific primer forward	ctgatccaaaacagcataacaattgagaggatgg
RT-qPCR NP segment-specific primer reverse	attcaagttggagtgccagatcatcaaatgggtgagaccag
